# Comparison of rhenium–porphyrin dyads for CO_2_ photoreduction: photocatalytic studies and charge separation dynamics studied by time-resolved IR spectroscopy†
†Electronic supplementary information (ESI) available: NMR spectra, cyclic voltammograms, crystallographic data, fluorescence data, spectra from photoreactions and photocatalytic data. CCDC 1406000 and 1406001. For ESI and crystallographic data in CIF or other electronic format see DOI: 10.1039/c5sc02099a


**DOI:** 10.1039/c5sc02099a

**Published:** 2015-08-20

**Authors:** Christopher D. Windle, Michael W. George, Robin N. Perutz, Peter A. Summers, Xue Zhong Sun, Adrian C. Whitwood

**Affiliations:** a Department of Chemistry , University of York , Heslington , York , YO10 5DD , UK . Email: robin.perutz@york.ac.uk; b School of Chemistry , University of Nottingham , Nottingham , NG7 2RD , UK . Email: mike.george@nottingham.ac.uk; c Department of Chemical and Environmental Engineering , The University of Nottingham Ningbo China , Ningbo , 315100 , China

## Abstract

We report a study of the photocatalytic reduction of CO_2_ to CO by zinc porphyrins covalently linked to [Re^I^(2,2′-bipyridine)(CO)_3_L]^+/0^ moieties with visible light of wavelength >520 nm. **Dyad 1** contains an amide C_6_H_4_NHC(O) link from porphyrin to bipyridine (Bpy), **Dyad 2** contains an additional methoxybenzamide within the bridge C_6_H_4_NHC(O)C_6_H_3_(OMe)NHC(O), while **Dyad 3** has a saturated bridge C_6_H_4_NHC(O)CH_2_; each dyad is studied with either L = Br or 3-picoline. The syntheses, spectroscopic characterisation and cyclic voltammetry of **Dyad 3 Br** and **[Dyad 3 pic]OTf** are described. The photocatalytic performance of **[Dyad 3 pic]OTf** in DMF/triethanolamine (5 : 1) is approximately an order of magnitude better than **[Dyad 1 pic]PF_6_** or **[Dyad 2 pic]OTf** in turnover frequency and turnover number, reaching a turnover number of 360. The performance of the dyads with Re–Br units is very similar to that of the dyads with [Re–pic]^+^ units in spite of the adverse free energy of electron transfer. The dyads undergo reactions during photocatalysis: hydrogenation of the porphyrin to form chlorin and isobacteriochlorin units is detected by visible absorption spectroscopy, while IR spectroscopy reveals replacement of the axial ligand by a triethanolaminato group and insertion of CO_2_ into the latter to form a carbonate. Time-resolved IR spectra of **[Dyad 2 pic]OTf** and **[Dyad 3 pic]OTf** (560 nm excitation in CH_2_Cl_2_) demonstrated electron transfer from porphyrin to Re(Bpy) units resulting in a shift of *ν*(CO) bands to low wavenumbers. The rise time of the charge-separated species for **[Dyad 3 pic]OTf** is longest at 8 (±1) ps and its lifetime is also the longest at 320 (±15) ps. The TRIR spectra of **Dyad 1 Br** and **Dyad 2 Br** are quite different showing a mixture of ^3^MLCT, IL and charge-separated excited states. In the case of **Dyad 3 Br**, the charge-separated state is absent altogether. The TRIR spectra emphasize the very different excited states of the bromide complexes and the picoline complexes. Thus, the similarity of the photocatalytic data for bromide and picoline dyads suggests that they share common intermediates. Most likely, these involve hydrogenation of the porphyrin and substitution of the axial ligand at rhenium.

## Abstract 2

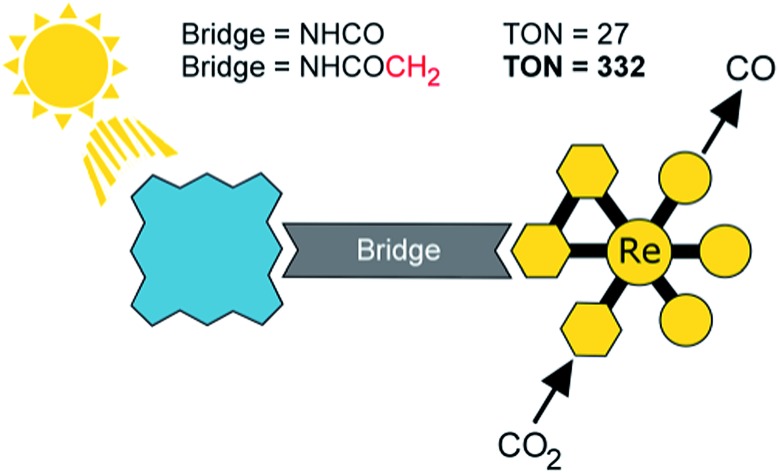
A new dyad for photocatalytic CO_2_ reduction produces ten times more CO and much longer-lived charge-separation than earlier rhenium-porphyrin dyads.

## Introduction

Much of the world's energy need is satisfied by the combustion of fossil fuels. The processes associated with generating energy in this way release gigatons of CO_2_ into the atmosphere every year, contributing to climate change.^1^ In addition to environmental unsustainability, the fossil fuels are essentially finite as they require geological timescales to form. The sun provides a clean source of energy that can satisfy our energy demands now and in the future.^2^ It is critical therefore, that we develop systems that can harvest visible light and store the energy as chemical fuel: systems that perform artificial photosynthesis.

Supramolecular assemblies containing components capable of light harvesting and catalysis can in principle perform artificial photosynthesis. There are several examples of this type of system for water oxidation,^3^,^4^ proton reduction,^5^–^9^ and CO_2_ reduction.^10^–^12^ For supramolecular assemblies to be active for photocatalytic redox reactions, they must be designed such that photoinduced electron transfer is favourable and such that charge separation lifetimes are sufficiently long for the catalytic reaction to occur prior to recombination.

Photocatalytic CO_2_ reduction to CO is an attractive choice because CO_2_ is consumed and CO can subsequently be converted into energy-dense hydrocarbon fuels.^13^–^16^ CO is also an industrial feedstock and a fuel in its own right.^17^ Diimine complexes of rhenium have received much attention since the discovery, reported in 1983, that they are active and selective photo- and electro-catalysts for CO_2_ reduction to CO.^18^ In the context of solar fuels, the rhenium complexes are limited because they cannot utilize much of the solar spectrum and turnover numbers of CO (TON_CO_) are low due to catalyst instability.^19^ Introduction of a sensitizer molecule can improve visible light absorption. The use of lower energy radiation and transferring the role of light absorption to another molecular unit will remove pathways of photo-degradation for the rhenium complex and increase stability. Indeed high TON_CO_ have been reported for dyads consisting of rhenium catalysts covalently linked to ruthenium bipyridyl units.^10^,^20^–^31^ Sensitizing dyes have also been used in association with Re catalysts supported on TiO_2_.^32^

Zinc porphyrins are good candidates for sensitization for several reasons.^33^ They show intense absorption in the visible spectrum, in particular the Q bands centred around 560 nm.^11^ The excited state redox potential of zinc porphyrin can be tuned to be negative with respect to the ground state of rhenium diimine complexes.^34^ The porphyrin motif is closely related to chlorophylls^35^ that are utilized in natural photosynthesis for light harvesting and charge separation.^36^ The visible light absorption and photoinduced electron-transfer ability of zinc porphyrins has led to high efficiencies in dye-sensitized solar cells.^37^ We and others recently demonstrated that zinc porphyrins can sensitize rhenium diimine complexes for CO_2_ reduction to CO with long-wavelength visible light.^11^,^38^,^39^

Rhenium bipyridine tricarbonyl complexes have been used extensively for photocatalytic and electrocatalytic CO_2_ reduction.^18^,^40^–^50^ There have been important recent developments in understanding the mechanism of such reactions. Kubiak has tracked reduced intermediates and their reactivity toward CO_2_.^45^,^46^,^51^,^52^ Ishitani has shown that the usual sacrificial reducing agent, triethanolamine (TEOA), coordinates to rhenium by deprotonation to form a rhenium alkoxide of the type ReOCH_2_CH_2_N(CH_2_CH_2_OH)_2_ which can insert CO_2_ to form a rhenium carbonate derivative.^53^ Inoue *et al.* have used mass spectrometry to study reduction of ReCl(4,4′dimethyl-2,2′-bipyridine)(CO)_3_ with triethylamine.^54^ They demonstrate that CO_2_ displaces a solvent molecule in the one-electron reduced complex to form a Re–CO_2_ radical which is then protonated to form a Re–COOH radical cation. Thus there is good evidence of direct CO_2_ coordination in the absence of TEOA and a complete cycle has been postulated for the electrochemical reaction.^52^ For the photochemical reaction with TEOA, the new evidence indicates CO_2_ insertion into the alkoxide complex, but the subsequent steps remain undefined.

Closely related zinc porphyrins bound to rhenium carbonyls have been investigated for photo-induced charge separation.^55^,^56^ Iron porphyrins have also been used successfully as electrocatalysts for CO_2_ reduction.^57^–^59^

There are several photophysical investigations into porphyrins linked to metal carbonyl complexes,^38^,^60^–^66^ but investigations connecting photophysical data and photocatalytic activity across a range of catalyst structures are scarce.^10^,^20^ Pump-probe time resolved infrared spectroscopy (TRIR) is an invaluable technique for measuring excited state dynamics in this kind of assembly.^34^,^67^–^80^ Metal carbonyl *ν*(CO) stretches can be observed with high intensity in a region of the infrared where few other vibrational bands are present. Crucially, they are very sensitive to the electron density on the metal centre and can be used to monitor charge transfer.

**Fig. 1:**
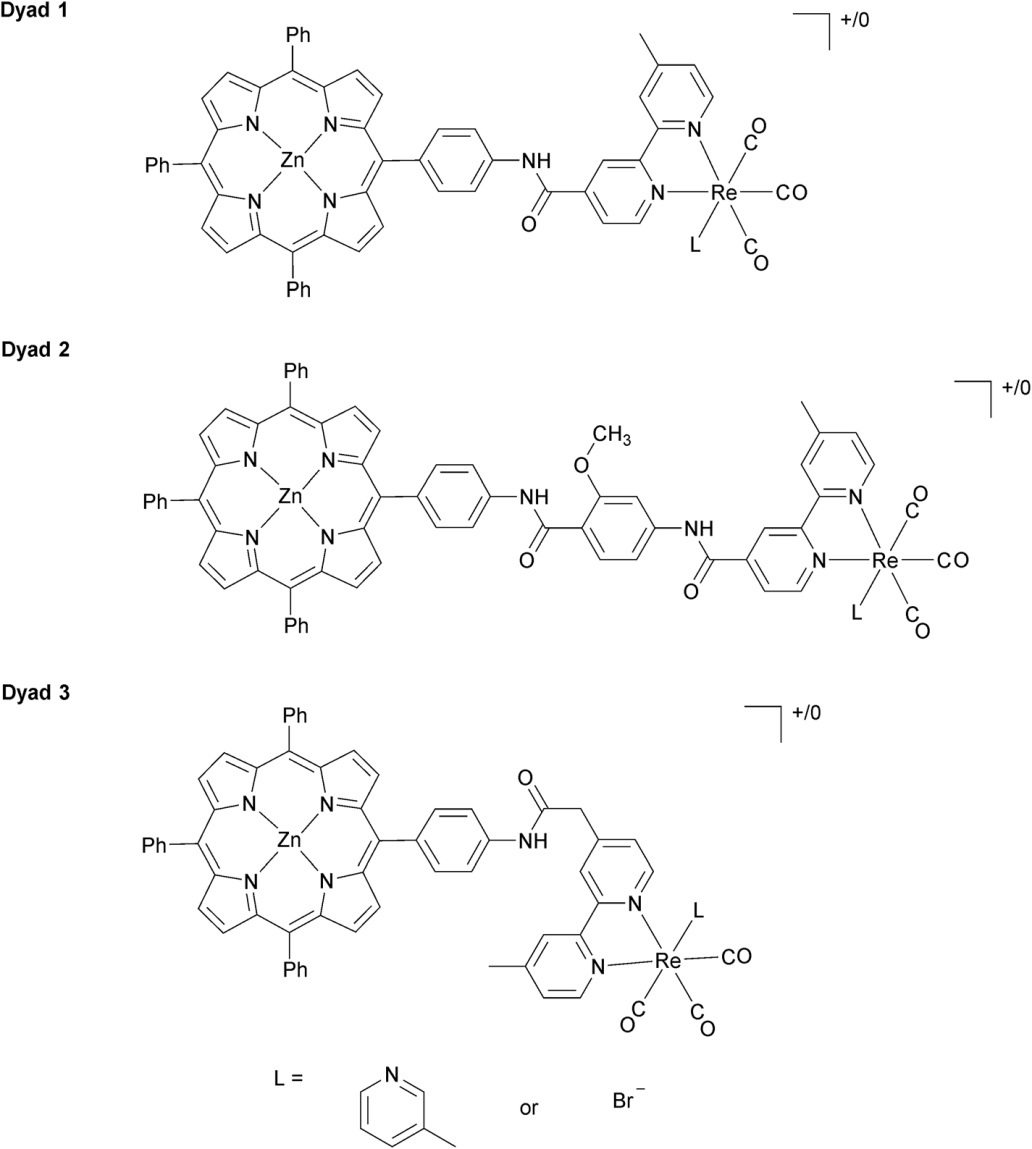
Structure of **Dyads 1–3**. When L = 3-picoline, the dyads are positively charged, and labelled **[Dyad 1 pic]PF_6_**, **[Dyad 2 pic]OTf**, and **[Dyad 3 pic]OTf**. The Br complexes are neutral and are labelled **Dyad 1 Br**, **Dyad 2 Br**, and **Dyad 3 Br**.

In our previous investigations of long-wavelength (*λ* > 520 nm) photocatalytic CO_2_ reduction with Re complexes covalently linked to zinc porphyrins, we investigated **[Dyad 1 pic]PF_6_** (Fig. 1) with a C_6_H_4_NHCO bridge.^11^ To increase catalytic activity we sought to reduce the rate of charge recombination by increasing the separation between donor and acceptor^74^,^75^ by inclusion of a methoxybenzamide molecular spacer (**[Dyad 2 pic]OTf**), and this dyad indeed displayed higher catalytic activity.^11^ We now report the synthesis and catalytic activity of a new dyad with a C_6_H_4_NHCOCH_2_ saturated molecular spacer **[Dyad 3 pic]OTf** (Fig. 1). We also compare the catalytic performance of these three cationic dyads to those of the corresponding neutral bromide complexes **Dyad 1 Br**, **Dyad 2 Br** and **Dyad 3 Br**. To our surprise the catalytic performance of each of the bromide complexes is very similar to that of the corresponding cationic dyads. This is intriguing as the bromide dyads do not undergo photoinduced reaction with intermolecular electron donors and their reduction potentials are significantly more negative than those of the cationic complexes.^81^,^82^

In our previous investigations of **[Dyad 1 pic]OTf** we showed by TRIR spectroscopy that charge separation occurs within a few ps and the lifetime of the charge-separated state is of the order of tens of ps. We now report on the TRIR spectroscopy of **[Dyad 2 pic]OTf**, **[Dyad 3 pic]OTF** and that of all three bromide complexes. We also show by TRIR spectroscopy that the excited state behaviour of the neutral bromide complexes is very different from that of the cationic picoline complexes. We propose mechanisms that can reconcile the different excited state and electrochemical behaviour with the similar photocatalysis.

## Experimental section

### General procedures

Chemicals were obtained from the following suppliers: diisopropylamine, 2.5 M *n*-butyl lithium in hexanes (Acros); EDTA, AgOTf, 4,4′-dimethyl-2,2′-bipyridine, sodium sulfide, celite 512 medium, Et_3_N, 2-chloro-methylpyridinium iodide, triethanolamine, anhydrous DMF, methyl chloroformate, copper(ii) acetate (Aldrich); 3-picoline (BDH Chemicals); CO_2_ – CP-grade with 5% CH_4_ (or 1% CH_4_) (BOC); Na_2_SO_4_, Na_2_CO_3_, NaHCO_3_, NaOH, HCl, KOH, ammonium hydroxide (Fisher); Zn(OAc)_2_·H_2_O, CH_3_CO_2_Na (Fisons).

Solvents for general use were obtained from Fisher. Solvents were dried by refluxing over sodium wire (C_6_H_6_, THF, toluene) or over CaH_2_ (CH_2_Cl_2_). DMF was dried using a Pure Solv 400-3-MD (Innovative Technology). For TRIR experiments, CH_2_Cl_2_ (99.9%, Merck) was distilled under an inert atmosphere of Ar from calcium hydride and anhydrous THF (≥99.9%, inhibitor-free, Sigma Aldrich) was used as supplied and stored in a glove box.

CD_2_Cl_2_, CD_3_OD, DMSO-*d*_6_ and CDCl_3_ were used as obtained (Aldrich) and THF-*d*_8_ was dried over potassium. Diisopropylamine was distilled from sodium hydroxide. Methyl chloroformate was distilled prior to use. *n*-BuLi was titrated against *n*-benzylbenzamide prior to use. Routine separation of porphyrins by flash chromatography was performed on a CombiFlash Rf system using 24 g RediSep Rf silica columns (Teledyne Isco), and dry-loading the samples on silica (Fluka).

#### NMR spectroscopy

NMR spectra were run on a Bruker AV500 (^1^H at 500 MHz) spectrometer or Bruker ECS400 (400 MHz). ^1^H NMR spectra were referenced to residual protiated solvent at *δ* 7.26 (CDCl_3_), *δ* 1.72 ([^2^H_8_] THF), *δ* 5.32 (CD_2_Cl_2_) and *δ* 3.31 (CD_3_OD).^83^^13^C{^1^H} NMR spectra were referenced to the solvent *δ* 77.16 (CDCl_3_), *δ* 25.31 ([^2^H_8_] THF), *δ* 53.84 (CD_2_Cl_2_) and *δ* 49.00 (CD_3_OD).^83^

#### IR and UV/vis absorption and emission

IR spectra were recorded on a Mattson RS FTIR instrument, averaging 64 scans at resolution 2 cm^–1^. ATR-IR spectra were an average of 32 scans. UV/visible absorption spectra were measured using an Agilent 8453 spectrometer. Steady state emission spectra were measured using a Hitachi F-4500 fluorimeter. The fluorescence was taken against a ZnTPP reference for the bromide complexes and against the individual dyad ligand ZnTPP-link-Bpy for the picoline complexes. Time-resolved emission was measured with an Edinburgh Instruments FLS980 equipped with a 560 nm pulsed LED (EPLED 560, pulsewidth 1.5 ns) and a red PMT detector. All samples were either degassed by three freeze–pump–thaw cycles or de-aerated by purging the sample with Ar. Correction was applied for instrument response. All absorption and emission measurements were made in 10 × 10 mm quartz cuvettes.

#### Mass spectrometry

ESI mass spectra were recorded on a Bruker micrOTOF instrument with a sample flow rate of 0.2 mL min^–1^, nebuliser gas pressure of 1.5 bar, dry gas flow of 8 L min^–1^ and a dry gas temperature of 180 °C. EI mass spectra were run on a Waters GCT premier with a source temperature of 180 °C, electron energy of 70 eV and a trap current of 200 μA. Some compounds and the reaction mixture ESI mass spectra were run on a Bruker Esquire 6000 *via* direct infusion using a syringe pump at 240 μL min^–1^. Nebuliser gas and dry gas flows and temperatures were optimised for each individual sample along with the spray voltage. *m*/*z* values are quoted for ^64^Zn, ^185^Re and ^79^Br.

#### Electrochemistry

Cyclic voltammetry was performed in CH_2_Cl_2_ with 0.1 M [Bu_4_N][PF_6_] (TBAP) electrolyte. The setup comprised reference electrode (Ag/AgCl, 3 M NaCl), working electrode (platinum disc) and counter electrode (platinum wire). Ferrocene was used as internal standard. All scans were made at 100 mV s^–1^. Cyclic voltammetric experiments used a BASi Epsilon potentiostat with C3 cell stand.

#### X-ray diffraction

X-ray diffraction data for **[Dyad 1 pic]PF_6_** and 5-[4-[(2-methoxy-4-nitro-phenylcarbonyl)-amino]phenyl]-10,15,20-triphenyl porphyrin were collected at 110 K on an Agilent SuperNova diffractometer with MoKα radiation (*λ* = 0.71073 Å). Data collection, unit cell determination and frame integration were carried out with “CrysalisPro”. Absorption corrections were applied using crystal face-indexing and the ABSPACK absorption correction software within CrysalisPro. Structures were solved and refined using Olex2 implementing SHELX algorithms. **[Dyad 1 pic]PF_6_** was solved using SUPERFLIP^84^ whereas 5-[4-[(2-methoxy-4-nitro-phenylcarbonyl)-amino]phenyl]-10,15,20-triphenyl porphyrin was solved using direct methods within the SHELXS algorithm. Structures were refined by full-matrix least squares using SHELXL-97. All non-hydrogen atoms were refined anisotropically. Carbon-bound hydrogen atoms were placed at calculated positions and refined using a “riding model”.

For **[Dyad 1 pic]PF_6_**, one of the phenyl groups on the porphyrin ring was disordered and modelled in two positions with refined occupancies of 0.817 : 0.183(12). The ADP of equivalent carbons in the disordered phenyl were constrained to be equal, *e.g.* C51 & C51A. The hexafluorophosphate was disordered over two sites. For one of these, the phosphorus was centred on a special position and for the other, the occupancy was 50% with a dichloromethane of crystallisation occupying the site at other times.

In addition to the ordered dichloromethanes of crystallisation, the crystal also contained some disordered solvent, believed to be a mix of hexane and dichloromethane for which a suitable discrete model could not be obtained. This was accounted for using a solvent mask; this space had a volume of 213 Å^3^ and predicted to contain *ca.* 17 electrons. The large residual density peaks are believed to provide evidence for twinning but a suitable method for modelling this was not found.

For 5-[4-[(2-methoxy-4-nitro-phenylcarbonyl)-amino]phenyl]-10,15,20-triphenyl porphyrin, the NH hydrogen was located by difference map. The crystal also contained dichloromethanes of crystallisation. One was partially occupied and was modelled with an occupancy of 0.1875; the carbon of this CH_2_Cl_2_ was restrained to be approximately isotropic. The other was fully occupied but disordered and modelled with the carbon in two different positions with relative occupancies of 0.814 : 0.186(12). Crystallographic parameters are listed in the ESI.†

#### Ultrafast infrared experiments

Picosecond time-resolved infrared (TRIR) spectra were obtained using purpose-built equipment based on a pump-probe approach. Details of the equipment and methods used for the TRIR studies have been described previously,^85^,^86^ a brief description of which is given here. The pump beam (560 nm, *ca.* 150 fs) and tunable probe beam (180 cm^–1^ spectral band width, *ca.* 150 fs) were generated from a commercial Ti:sapphire oscillator (MaiTai)/regenerative amplifier system (Spitfire Pro, Spectra Physics). The mid-IR probe was detected using a 128-element HgCdTe array detector (Infrared Associates) typically with a resolution of *ca.* 4 cm^–1^. All the solutions for analysis were prepared under an inert atmosphere of Ar, degassed by three freeze–pump–thaw cycles and put under Ar. **[Dyad 2 pic]OTf** and **[Dyad 3 pic]OTf** were run in CH_2_Cl_2_ at 1.5 mM and 1.0 mM respectively, with a path length of 0.5 mm. **Dyad 1 Br**, **Dyad 2 Br** and **Dyad 3 Br** were run in THF at 1 mM with a path length of 0.25 mm. A Harrick solution cell with CaF_2_ windows was used and 20 mL of solution was continuously circulated during the measurements.

#### Photocatalysis

Photocatalysis was performed in a custom-made cell^67^ comprised of a 10 × 10 mm quartz cuvette with a headspace of a minimum volume of 10 mL. Above the headspace was a ground glass joint, which was sealed with a size 21 septum. Samples were taken through this septum for GC analysis. The headspace had a sidearm, isolated by a Young's tap, joining it to a gas phase IR cell with CaF_2_ windows. The IR cell was connected to a vacuum joint *via* a second Young's tap. The IR cell was put under vacuum. At the end of a catalytic run the headspace was opened to the IR cell and the gas produced from the reaction would be drawn through and could be monitored by IR spectroscopy.

The concentration of catalytic solution was typically 0.05 mM, making the absorbance of the porphyrin Q band at 560 nm, Q(1, 0), *ca.* 1 by UV/vis spectroscopy. A 10 mL stock solution of 0.25 mM catalyst in DMF would typically be made. These stock solutions allowed the catalysts to be weighed out in amounts greater than 1 mg. They were stored in a freezer at –25 °C and could be used up to a month later without noticeable degradation in their catalytic performance, UV/vis spectrum or mass spectrometric analysis. The 0.05 mM catalytic solution was made from the stock by diluting 2 mL into 10 mL. To make a 10 mL solution in DMF : TEOA 5 : 1, 1.87 g TEOA was weighed into a 10 mL volumetric flask, approximately 2 mL of DMF was added so the catalytic stock was not being added to neat TEOA. Then 2 mL of stock was added, followed by DMF up to the 10 mL mark. The catalytic solutions were protected from light as much as possible and stored in the freezer. A sample (3 mL) of catalytic solution was added to the photoreaction cuvette and was bubbled with CO_2_/CH_4_ 95/5 for 10 min, protected from light throughout this time.

Irradiation of all samples was performed with an ILC 302 Xe arc lamp. Light from the lamp was directed through a water filter (10 cm) and a 660 nm short pass filter (<660 nm, Knight Optical) to remove heat, such that any sample directly in the beam was at a temperature of 33 °C. A *λ* > 520 nm optical filter was added (Schott).

The amount of CO produced was determined by GC analysis using a UnicamProGC+ (ThermoONIX) with a thermal conductivity detector. Air, CO, CH_4_ and CO_2_ were separated on a Restek ShinCarbonST 100/120 micropacked column (2 m, 1/16′′ OD, 1.0 mm ID) fitted with “pigtails” of Restek intermediate-polarity deactivated guard column on either end (fused silica, 0.53 mm ID, 0.69 ± 0.05 mm OD). The carrier gas was ultra high purity He (N6.0, BOC gases) passed through a GC triple filter (Focus Technical) to remove trace impurities prior to the column. The GC method began with 1 min at 40 °C followed by a 5 °C min^–1^ gradient up to 120 °C (16 min). Injections (200 μL) were made manually with a Hamilton gastight locking syringe (500 μL) at 220 °C with a 30 mL min^–1^ split flow. The carrier gas was kept at constant pressure (165 kPa). The detector block and transfer temperatures were 200 and 190 °C respectively, at a constant voltage of 10 V with makeup and reference flows of 29 and 30 mL min^–1^ respectively. The amount of CO was determined using a calibration plot. Known volumes of CO were mixed with a mimic experimental solution (3 mL DMF : TEOA 5 : 1 (v/v)), headspace and solution were purged with CO_2_ : CH_4_ (99 : 1 or 95 : 5) and sampled to GC. Quantification was by comparison of integrations of the CO peak against the CH_4_ internal standard. Corrections were made for temperature and the change in headspace pressure at each injection.

### Synthesis

Re(CO)_5_Br,^87^ 5-(4-aminophenyl)-10,15,20-triphenylporphyrin,^88^**Dyad 1 Br**, **[Dyad 1 pic]OTf**,^82^**Dyad 2 Br** and **[Dyad 2 pic]OTf**^11^ were synthesised by literature methods.

#### 4′-Methyl-2,2′-bipyridine-4-acetic acid (**AABpy**)

Procedure 1: a modification of that by Ciana.^89^ A 250 cm^3^ round-bottomed flask was flame dried and flushed with Ar. THF (3 cm^3^) and diisopropylamine (2.1 cm^3^) were added and the mixture cooled to –78 °C. 2.5 M butyl lithium in hexanes (6 cm^3^) was added *via* syringe and the mixture was stirred for 0.75 h. A solution of 4,4′-dimethyl bipyridine (3 g) in THF (72 cm^3^) was added, the solution turned black and was stirred for 2 h at –78 °C. Dry CO_2_ (g) was set bubbling through a flame dried round-bottomed flask charged with Et_2_O (30 cm^3^) and cooled to –78 °C. The black lithiated bipyridine solution was added to the Et_2_O/CO_2_ mixture *via* cannula and a yellow precipitate soon appeared. The reaction was left under an atmosphere of CO_2_ overnight and allowed to warm to RT. Et_2_O was added (30 cm^3^) and the product extracted with 3 M NaOH (3 × 30 cm^3^). The alkaline layer was then acidified to pH 1 with concentrated HCl and cooling. The product was then extracted with Et_2_O (30 cm^3^) and buffered to pH 5 with solid CH_3_CO_2_Na. A saturated aqueous solution of Cu(CH_3_CO_2_)_2_ was added causing precipitation of a blue Cu complex. The solid was filtered off with a microfiber filter paper and washed with water, ethanol and ether and then air-dried. The product was suspended in water (60 cm^3^) and H_2_S bubbled through for 20 min resulting in a dark brown colour. The product was filtered through celite, concentrated to 9 cm^3^ and filtered again. The solution was evaporated to dryness under reduced pressure to yield a yellow oil. Recrystallisation twice from ethanol/hexane yielded pure **AABpy** (569 mg, 2.682 mmol, 16%). Analysis was in agreement with the literature.^89^

Procedure 2: a modification of that by Tomioka.^90^ To a flame dried 100 mL round-bottomed flask was added THF (5 mL) and freshly distilled diisopropylamine. The mixture was cooled to –78 °C and freshly titrated *n*-butyl lithium (1.1 eq.) was added. Dimethylbipyridine (1 g, 5.43 mmol) was dissolved in THF (20 mL) and added by cannula. The mixture was stirred at –78 °C for 2 h and then freshly distilled methyl chloroformate (0.6 mL) in THF (2 mL) was added by syringe. The reaction was stirred at –78 °C for 1 h and then at RT for 2 h. The mixture was then washed with saturated NaHCO_3_ solution and extracted into ethyl acetate. The extracts were washed with brine and dried over Na_2_SO_4_. The product was purified on Si-60 eluting with 2% Et_3_N in pentane and 0–10% EtOAc. The second fraction was collected and the solvent removed (257 mg, 0.858 mmol, 22%).

^1^H NMR (400 MHz, CDCl_3_): *δ* 2.45 (3H, s, Bpy CH_3_); 3.72 (3H, s, OCH_3_); 3.73 (2H, s, CH_2_); 7.15 (1H, dd, *J* = 0.80, 5.03 Hz Bpy); 7.28 (1H, dd, *J* = 1.68, 5.06 Hz, Bpy); 8.24 (1H, s, Bpy); 8.33 (1H, s, Bpy); 8.54 (1H, d, *J* = 4.92 Hz, Bpy); 8.63 (1H, d, *J* = 5.01 Hz, Bpy).

The methyl ester was hydrolysed to produce the free acid. A 50 mL round-bottomed flask was charged with the methyl ester (284 mg), which was dissolved in the minimum amount of methanol. KOH (131 mg) was added. The reaction was stirred at 35 °C for 2 h. The solvent was removed and the solid taken up in H_2_O and titrated to pH 7 with a 10% solution of HCl. The H_2_O was removed and the product used without purification.

^1^H NMR (400 MHz, CD_3_OD): *δ* 2.43 (3H, s, Bpy CH_3_); 3.58 (2H, s, CH_2_); 7.24 (1H, dd, *J* = 0.73, 5.08 Hz, Bpy); 7.38 (1H, dd, *J* = 1.51, 5.08 Hz, Bpy); 8.03 (1H, s, Bpy); 8.17 (1H, s, Bpy); 8.45 (1H, d, *J* = 5.03 Hz, Bpy); 8.48 (1H, d, *J* = 5.08 Hz, Bpy).

#### 5-[4-(4-Methylene carboxyamidyl,4′-methyl-2,2′-bipyridine-)phenyl]-10,15,20-triphenyl porphyrin (**CH_2_Bpy-H_2_TPP**)

A 50 cm^3^ round-bottomed flask was charged with **NH_2_-H_2_TPP** (125 mg, 0.219 mmol) and CH_2_Cl_2_ (15 cm^3^) and cooled to 0 °C. A solution of **AABpy** and 2-chloromethyl pyridinium iodide in CH_2_Cl_2_ (15 cm^3^) was added, followed by Et_3_N dropwise. The mixture was stirred at 0 °C for 5 min and then warmed to RT. After stirring at RT for 0.5 h TLC showed negligible quantities of starting porphyrin and so the reaction was stopped. The reaction was quenched with 10% HCl (50 cm^3^) and the porphyrin extracted with CH_2_Cl_2_. The extract was washed with saturated NaHCO_3_ followed by brine and then dried over MgSO_4_. The product was purified with column chromatography on Si-60 eluting with CH_2_Cl_2_ and CH_3_OH (0% to 3%). The second fraction was collected and the solvent removed to yield the desired product (156 mg, 0.186 mmol, 94%).

^1^H NMR (400 MHz, CDCl_3_): *δ* 2.44 (3H, s, Bpy CH_3_); 3.90 (2H, s, CH_2_ spacer); 7.19 (1H, d, *J* = 4.46 Hz, Bpy 5′); 7.50 (1H, d, *J* = 4.80 Hz, Bpy 5); 7.70 (1H, s, Bpy 3); 7.77 (9H, m, *m*-/*p*-phenyl); 7.88 (2H, d, *J* = 8.02 Hz, *m*-amidophenyl); 8.16 (2H, d, *J* = 8.08 Hz, *o*-amidophenyl); 8.23 (6H, m, *o*-phenyl); 8.33 (1H, s, Bpy 3′); 8.53 (1H, s, amide); 8.60 (1H, d, *J* = 4.97 Hz, Bpy 6′); 8.78 (1H, d, *J* = 5.14 Hz, Bpy 6); 8.86 (8H, m, β-pyrrole).

ESI-MS: *m*/*z* = 840.3428 ([M + H^+^]^+^, 100%), (M + H^+^; C_57_H_42_N_7_O requires 840.3445, difference 1.7 mDa).

#### 5-[4-(4-Methylene carboxyamidyl,4′-methyl-2,2′-bipyridine-)phenyl]-10,15,20-triphenyl porphyrinatozinc(ii) (**CH_2_Bpy-ZnTPP**)

A 100 cm^3^ round-bottomed flask was charged with **CH_2_Bpy-H_2_TPP** (152 mg, 181 μmol), Zn(OAc)_2_ (179 mg, 815 μmol), CH_3_OH (5 cm^3^) and CHCl_3_ (25 cm^3^). The mixture was heated to reflux for 1 h and the reaction was followed by UV/vis spectroscopy. The reaction mixture was allowed to cool, pumped to dryness and re-dissolved in 100 cm^3^ CHCl_2_ and 20 cm^3^ CHCl_3._ This was washed with EDTA solution (2 g in 200 cm^3^ 10% Na_2_CO_3_ solution), water (3 × 200 cm^3^), dried (MgSO_4_) and the solvent removed to yield the desired compound (160 mg, 178 μmol, 98%).

^1^H NMR (400 MHz, THF-*d*_8_): *δ* 2.49 (3H, s, Bpy CH_3_); 3.95 (2H, s, CH_2_); 7.21 (1H, d, *J* = 4.49 Hz, Bpy 5′); 7.55 (1H, d, *J* = 4.08 Hz, Bpy 5); 7.78 (9H, m, *m*-/*p*-phenyl); 8.09 (2H, d, *J* = 8.43 Hz, *m*-amidophenyl); 8.14 (2H, d, *J* = 8.43 Hz, *o*-amidophenyl); 8.23 (6H, m, *o*-phenyl); 8.46 (1H, s, Bpy 3′); 8.56 (1H, d, 4.89 Hz, Bpy 6′); 8.67 (1H, d, *J* = 5.03 Hz, Bpy 6); 8.68 (1H, s, Bpy 3); 8.86 (6H, m, β-pyrrole); 8.92 (2H, d, *J* = 4.62 Hz, β-pyrrole); 9.73 (1H, s, amide).

ESI-MS: *m*/*z* = 902.2556 ([M + H^+^]^+^, 100%), (M + H^+^ requires 902.2580, difference 2.4 mDa).

#### 5-{4-[Rhenium(i)tricarbonyl(bromide)-4-methyl-2,2′-bipyridine-4′-methylene carboxyamidyl]phenyl}-10,15,20-triphenylporphyrinatozinc(ii) (**Dyad 3 Br**)

A two-neck 50 cm^3^ round-bottomed flask was fitted with a reflux condenser and gas valve. The setup was flame dried. Under Ar **CH_2_Bpy-ZnTPP** (200 mg, 221 μmol) was added, followed by ReBr(CO)_5_ (90 mg, 221 μmol). Dry benzene was added (30 cm^3^) by syringe. The mixture was heated to 65 °C and the reaction was followed by IR spectroscopy and judged to be complete after 22 h. The reaction mixture was filtered to leave a solid product and used without further purification (263 mg, 210 μmol, 95%).

^1^H NMR (500 MHz, THF-*d*_8_): *δ* 2.60 (3H, s, Bpy methyl); 4.09 (2H, s, methylene); 7.47 (1H, dd, *J* = 0.68, 5.61 Hz, Bpy-5′); 7.73 (10H, m, *m*-, *p*-phenyl + Bpy-5); 8.05 (2H, d, *J* = 8.61 Hz, bridging phenyl); 8.13 (2H, d, *J* = 8.37 Hz, bridging phenyl); 8.18 (6H, m, *o*-phenyl); 8.45 (1H, s, Bpy-3′); 8.64 (1H, d, *J* = 0.84 Hz, Bpy-3); 8.82 (6H, m, β-pyrrole); 8.86 (2H, m, β-pyrrole); 8.90 (1H, d, *J* = 5.69 Hz, Bpy-6′); 9.03 (1H, d, *J* = 5.69 Hz, Bpy-6); 9.82 (1H, s, amide).

^13^C{^1^H} NMR (400 MHz, THF-*d*_8_): *δ* 21.12 (Bpy CH_3_); 43.48 (methylene); 117.72 (*m*-amidophenyl); 120.86 (porphyrin *meso* by amidophenyl); 121.26 (porphyrin *meso*); 125.05 (Bpy-3′); 125.20 (Bpy-3); 126.96 (*m*-phenyl); 127.88 (i-phenyl); 128.48 (Bpy-6′); 128.63 (Bpy-6); 128.81 (Bpy-4); 131.97 (β-pyrrole); 135.13 (*o*-phenyl); 135.47 (*o*-amidophenyl); 139.48 (i-amidophenyl); 139.62 (*p*-amidophenyl); 144.31 (*p*-phenyl); 149.60 (Bpy-4′); 150.76 (β-pyrrole); 152.41 (Bpy-5); 153.22 (Bpy-5′); 153.48 (Re carbonyl); 156.41 (Bpy-2); 156.60 (Bpy-2′); 167.22 (amide carbonyl); 198.49 (Re carbonyl).

IR (*ν*/cm^–1^) (THF) 2019, 1919, 1895 (*ν*(CO)). (ATR) 2021 (CO), 1935 (CO), 1892 (CO), 1668 (C=O), 1622, 1595, 1525 (N–H deformation), 1484, 1441, 1398, 1341, 1241, 1206, 1186, 1070, 993, 828, 797, 756, 717, 703, 686.

ESI-MS: *m*/*z* = 1248.9 ([M + H^+^]^+^, 23%), (M + H^+^; C_60_H_40_N_7_O_4_ZnReBr requires 1249.1).

#### 5-{4-[Rhenium(i)tricarbonyl(3-picoline)-4-methyl-2,2′-bipyridine-4′-methylene carboxyamidyl]phenyl}-10,15,20-triphenylporphyrinatozinc(ii) trifluoromethanesulfonate (**[Dyad 3 pic]OTf**)

A two-neck 50 cm^3^ round-bottomed flask was fitted with a gas valve and flame dried. It was taken into a glovebox and AgOTf was added (206 mg, 800 μmol). A condenser fitted with a single-neck round-bottomed flask and gas valve was flame dried, then the round-bottomed flask was removed under Ar and the condenser and reaction flask were brought together. THF and 3-picoline (1.09 mL, 11.2 mmol) were added and finally **Dyad 3 Br** (200 mg, 160 μmol). The mixture was heated to reflux for 2 h and checked for completion by IR spectroscopy. The mixture was allowed to cool, filtered to remove AgBr and dried under vacuum for 72 h. The oil was re-dissolved in THF and applied to Sephadex LH20 eluting with THF. The THF was removed and the solid washed with an ethanol/petrol 20/80 mixture. The solid was dried to yield **[Dyad 3 pic]OTf** (95 mg, 67.10 μmol, 42%).

^1^H NMR (400 MHz, THF-*d*_8_): *δ* 2.26 (3H, s, picoline methyl); 2.68 (3H, s, Bpy methyl); 4.27 (2H, s, methylene); 7.31 (1H, d, *J* = 5.73, 8.08 Hz, pic); 7.71 (1H, d, *J* = 5.91 Hz Bpy); 7.74 (1H, d, *J* = 7.88 Hz, pic); 7.79 (9, m, *m*-, *p*-phenyl); 8.15 (3H, m, bridging phenyl + Bpy); 8.26 (9H, m, *o*-phenyl + pic); 8.34 (1H, s, pic); 8.89 (6H, m, β-pyrrole); 8.95 (2H, m, β-pyrrole); 9.01 (1H, s, Bpy); 9.17 (1H, d, *J* = 5.60 Hz, Bpy); 9.22 (1H, s, Bpy); 9.33 (1H, d, 5.75 Hz, Bpy); 10.44 (1H, s, amide).

^13^C{^1^H} NMR (400 MHz, THF-*d*_8_): *δ*_C_ 17.86 (picoline CH_3_); 21.14 (Bpy CH_3_); 44.30 (methylene); 117.94 (*m*-amidophenyl); 121.22 (porphyrin *meso*); 126.93 (*m*-phenyl + Bpy-3); 127.18 (picoline-5); 127.87 (*p*-phenyl); 129.86 (Bpy-5); 130.23 (Bpy-5′); 131.97 (β-pyrrole); 135.20 (*o*-phenyl + *o*-amidophenyl); 138.09 (picoline-3); 139.23 (i-amidophenyl); 139.84 (*p*-amidophenyl); 141.13 (picoline-4); 144.41 (i-phenyl); 149.78 (picoline-6); 150.76 (β-pyrrole); 151.07 (β-pyrrole); 152.70 (Bpy-4′); 153.22 (Bpy-6); 153.61 (Bpy-6); 155.40 (Bpy-4); 156.73 (Bpy-2); 156.97 (Bpy-2′); 167.22 (amide carbonyl); 192.50 (Re carbonyl); 196.68 (Re carbonyl).

IR (*ν*/cm^–1^) (CH_2_Cl_2_) 2034, 1933, 1924 (*ν*(CO)) (ATR) 2029 (CO), 1912 (CO), 1676 (C=O + N–H), 1597, 1522 (N–H deformation), 1486, 1340, 1280, 1245, 1158, 1068, 1027, 993, 796, 702.

ESI-MS: *m*/*z* = 1265.2453 (M^+^, 100%), (M^+^; C_66_H_46_N_8_O_4_ZnRe requires 1265.2476 difference 2.3 mDa).

## Results

### Synthetic methodology

**Fig. 2:**
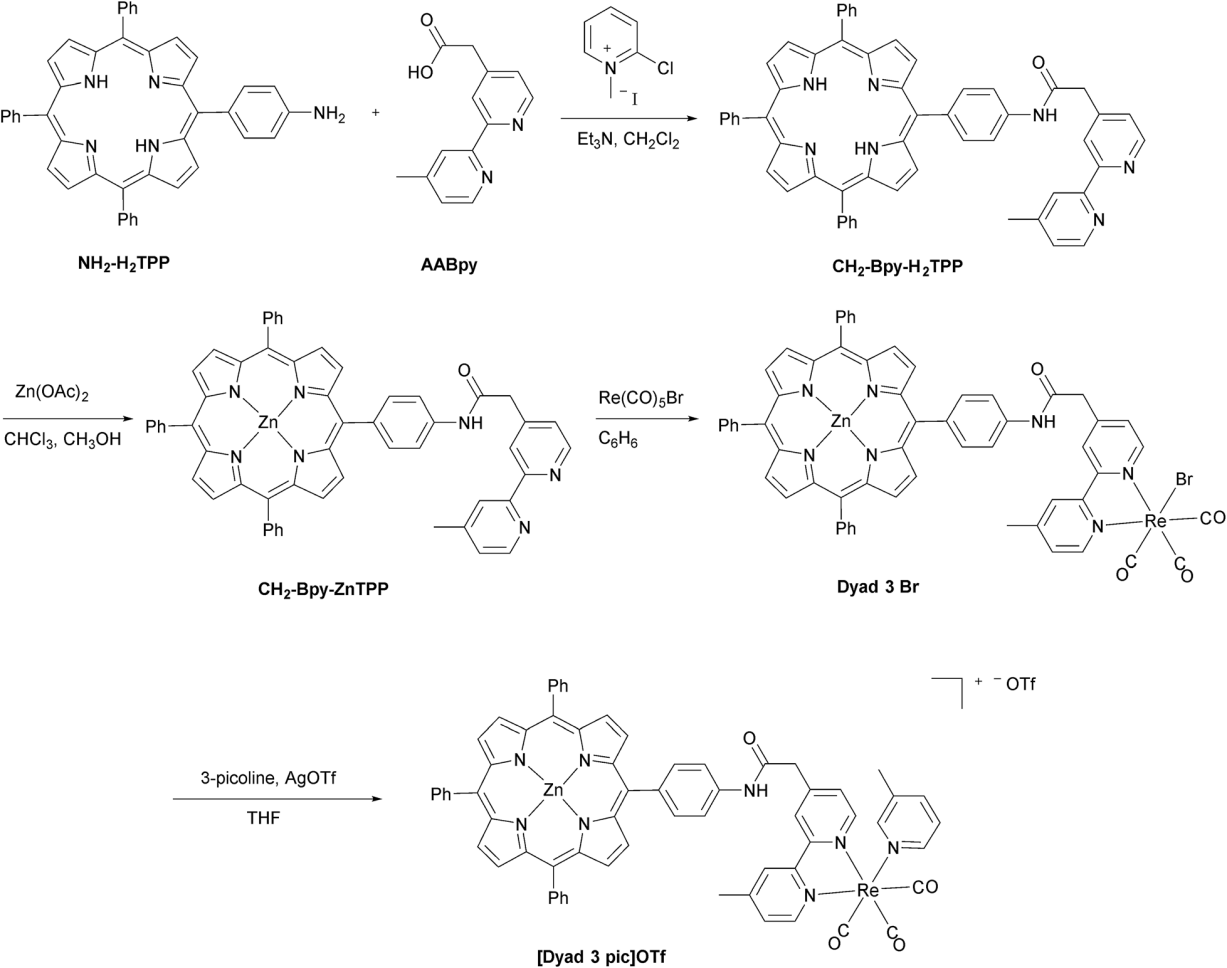
Preparation of **[Dyad 3 pic]OTf**.

The synthetic methods for the preparation of **[Dyad 1 pic]OTf**, **Dyad 1 Br**, **[Dyad 2 pic]OTf** and **Dyad 2 Br** have been reported previously.^11^,^34^ The synthetic strategy for **[Dyad 3 pic]OTf** is shown in Fig. 2. **AABpy**^89^ and **NH_2_-H_2_TPP**^88^ were prepared by literature procedures. The two were coupled using 2-chloro-methylpyridinium iodide in excellent yield (94%).^91^ Zinc was inserted into the porphyrin and Re(CO)_3_Br was complexed to the Bpy as reported previously for **[Dyad 1 pic]OTf**.^34^ Bromide was substituted for 3-picoline using AgOTf in THF. The product was purified using size exclusion chromatography (Sephadex LH20) eluting with THF.

**Dyad 3 Br** and **[Dyad 3 pic]OTf** were characterized using ^1^H and ^13^C{^1^H} NMR spectroscopy, mass spectrometry, UV/vis spectroscopy and IR spectroscopy. Both ^1^H and ^13^C NMR signals were assigned with the aid of COSY and NOE experiments (Fig. S1–S11†). The ESI mass spectrum of **[Dyad 3 pic]OTf** is shown in Fig. S12.† The UV/vis spectra were dominated by the porphyrin, which in the ground state was unaffected by the rhenium unit, displaying a typically sharp and intense Soret band at 420 nm and three less intense Q-bands at 510, 548 and 588 nm. IR spectra of the metal carbonyl region were consistent with those of ReBpy(CO)_3_L complexes, displaying three stretches (2019, 1919, 1895 cm^–1^; THF) for **Dyad 3 Br** indicating *C*_s_ symmetry. **[Dyad 3 pic]OTf** showed one sharp and one broad (2034, 1933–1924 cm^–1^; CH_2_Cl_2_) stretch, indicating pseudo *C*_3v_ symmetry. The signals of the picoline complex are at higher wavenumber than those of the bromide, consistent with a cationic rhenium centre.

### Cyclic voltammetry

The electrochemical behaviour of **[Dyad 1 pic]PF_6_**, **Dyad 1 Br** and **[Dyad 2 pic]OTf** have been reported previously.^11^,^82^ Cyclic voltammograms of **[Dyad 3 pic]OTf** were run in CH_2_Cl_2_ (Fig. S13†). Two reversible oxidation waves were observed on scanning to anodic potentials, corresponding to the first and second oxidation of the porphyrin.^92^ In the cathodic direction a quasi-reversible reduction wave was observed, corresponding to the first reduction of the rhenium unit. The first oxidation of the porphyrin is at similar potential to that of **[Dyad 2 pic]OTf** and the Re-free porphyrin **CH_2_-Bpy-ZnTPP**, but the reduction of the rhenium is at a more negative potential than those of either **[Dyad 1 pic]PF_6_** or **[Dyad 2 pic]OTf** (Table 1).^11^ This is probably due to the +I effect of the CH_2_ moiety and the separation that it provides from the electron-withdrawing carboxamide group. Cyclic voltammograms of **Dyad 3 Br** in CH_2_Cl_2_ displayed two porphyrin-based reversible oxidations (Fig. S14†). The first oxidation of the porphyrin is at 40 mV more positive potential than that of **[Dyad 3 pic]OTf**. Its potential is the same as **Dyad 1 Br** but is at 70 mV more positive potential than in **Dyad 2 Br**. The cyclic voltammogram displays an irreversible peak in the cathodic direction, assignable to the first reduction of the rhenium unit. This peak is 150 mV more negative than that of **[Dyad 3 pic]OTf** and ≥200 mV more negative than those of **Dyad 1 Br** and **Dyad 2 Br**. Based on the ratio of peak currents, the reduction of **Dyad 3 Br** is irreversible whereas the reduction of **[Dyad 3 pic]OTf** is quasi-reversible.

**Table 1:** First oxidation and first reduction potentials in CH_2_Cl_2_ (*vs.* Fc/Fc^+^)

Dyad	*E* ox 1/2 /V	*E* red 1/2 /V
**[Dyad 1 pic]PF_6_** ^*a*^	0.28	–1.44
**Dyad 1 Br**	0.36	–1.63
**[Dyad 2 pic]OTf** ^*a*^	0.32	–1.42
**Dyad 2 Br**	0.30	–1.60
**[Dyad 3 pic]OTf**	0.33^*b*^	–1.68
**Dyad 3 Br**	0.37^*b*^	–1.83

^*a*^From ref. 11.

^*b*^The CV of the Re-free porphyrin **CH_2_-Bpy-ZnTPP** gave *E*ox1/2 = 0.32 V, a shift of 60 mV to lower potential with respect to ZnTPP. Corresponding measurements on **Bpy-ZnTPP** gave a shift of 50 mV with respect to ZnTPP in THF.^34^

### X-ray crystallography

**Fig. 3:**
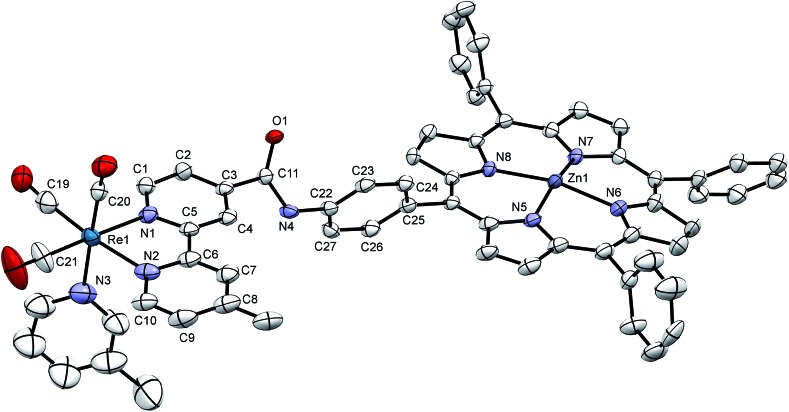
X-ray crystal structure of **[Dyad 1 pic]PF_6_** showing the asymmetric unit. Hydrogen atoms and disorder at one phenyl omitted for clarity. Thermal ellipsoids shown with probability of 50%. Two asymmetric units are linked head-to-tail to form a dimer bound through Zn–O(1) bonds (see Fig. S15†).

Crystals were grown of **[Dyad 1 pic]PF_6_** by layering hexane onto a CH_2_Cl_2_ solution of the complex. The crystal structure (Fig. 3) suffers from disorder, but nevertheless establishes some key parameters. The zinc atom sits out of the porphyrin ring by 0.2568(6) Å. As is typical for tetraphenyl porphyrins,^67^ the phenyl groups are twisted with respect to the plane of the porphyrin. The C_6_H_4_ ring joined to the amide is 65.7(3)° out of the plane of the porphyrin. The C_6_H_4_ ring is 70.8(3)° out of the plane of the Bpy making the Bpy almost coplanar with the porphyrin, separated by only 9.15(14)°. The two rings of the Bpy are not quite coplanar as there is a twist of 8.3(3)°. Only on acquiring X-ray data was it possible to identify that the compound exists as a dimer (Fig. S15†) resulting in a pair of equivalent weak Zn–O bonds (O(1)–Zn 2.216(4) Å) that bind an amido oxygen in one ion to the zinc on the adjacent partner ion and *vice versa*. Crystals were also grown of the **[Dyad 2 pic]OTf** precursor 5-[4-[(2-methoxy-4-nitro-phenylcarbonyl)-amino]phenyl]-10,15,20-triphenyl porphyrin (Fig. S16†). The structure confirms that the hydrogen atom on the porphyrin amide forms a hydrogen bond with the oxygen of the methoxy group, consistent with the low field at which the amide proton resonates in the ^1^H NMR spectrum.^11^

### Emission spectroscopy

Comparison of the singlet ππ* fluorescence from the porphyrin moiety of a dyad, with that of a suitable rhenium-free analogue provides valuable information on the quenching ability of the rhenium unit. We have previously reported emission quenching determined in this way for the zinc porphyrin unit in **[Dyad 1 pic]OTf** (>95% in PrCN) and for **Dyad 1 Br** (50% in THF) compared with the emission of the rhenium-free ZnTPP-link-Bpy analogue.^34^,^81^ Similar emission measurements were performed with **[Dyad 2 pic]OTf** and **[Dyad 3 pic]OTf** (Table 2) showing 55% and 23% emission quenching, respectively (Fig. S17 and S18†). The emission lifetimes (Table 2) show a similar trend to the steady state measurements. **[Dyad 1 pic]PF_6_** has the shortest lifetime, followed by **[Dyad 2 pic]OTf**, which also shows a longer component. **[Dyad 3 pic]OTf** shows the least shortening of the fluorescence lifetime (Fig. S19†). The emission quenching is attributed principally to electron transfer from excited state zinc porphyrin to the rhenium with only minor heavy atom effects. The large variation in quenching is suggestive of corresponding variations in electron transfer rates.

**Table 2:** Photophysical data for **Bpy-ZnTPP** reference compound and all dyads^*a*^

Compound	Solvent	Steady state quenching (%)	*φ*_f_ ^*b*^	*τ*_1_, *τ*_2_^*b*^ (ns)	*σ* ^*b*^ (ns)	*k*_Q_ ^*b*^ (ns^–1^)
**Bpy-ZnTPP**	CH_2_Cl_2_		0.033^*c*^	1.72	2 × 10^–3^	—
**[Dyad 1 pic]PF_6_**	CH_2_Cl_2_	95^*d*^ ^,^^*e*^	0.0017	0.024^*g*^	—	—
**[Dyad 2 pic]OTf**	CH_2_Cl_2_	55^*e*^	0.015	0.69 (88%), 1.65 (12%)	1 × 10^–2^, 7 × 10^–2^	0.87
**[Dyad 3 pic]OTf**	CH_2_Cl_2_	23^*e*^	0.025	1.56	3 × 10^–3^	0.06
**Bpy-ZnTPP**	THF		0.033	1.80	2 × 10^–3^	
**Dyad 1 Br**	THF	41^*f*^	0.019	0.97	2 × 10^–2^	0.48
**Dyad 2 Br**	THF	11^*f*^	0.029	1.66	2 × 10^–3^	0.05
**Dyad 3 Br**	THF	0^*f*^	0.033	1.81	2 × 10^–3^	0

^*a*^ *λ*_ex_ = 560 nm, *λ*_em_ = 605 nm.

^*b*^ *φ*_f_ = fluorescence quantum yield, *τ* emission lifetime with standard deviation *σ*, *k*_Q_ = *τ*_dyad_^–1^ – *τ*_ref_^–1^.

^*c*^Value for ZnTPP in toluene, from ref. 93.

^*d*^ **[Dyad 1 pic]OTf** from ref. 34.

^*e*^Emission yields relative to rhenium-free Bpy-link-TPP analogue.

^*f*^Relative to ZnTPP; note that yield of **CH_2_-Bpy-ZnTPP** is *ca.* 15% greater than that of ZnTPP.

^*g*^Lifetime was shorter than the instrument response time. This value is taken from time-resolved absorption from ref. 34.

Emission quenching in **Dyad 1 Br**, **Dyad 2 Br** and **Dyad 3 Br** was measured relative to zinc tetraphenylporphyrin in THF (Fig. S20†). In agreement with previous reports,^81^**Dyad 1 Br** displays 41% emission quenching relative to a simple zinc porphyrin while **Dyad 2 Br** and **Dyad 3 Br** show 11% and 0% emission quenching, respectively. A very similar trend is observed in the emission lifetimes (Table 2, Fig. S21†). We also checked the emission yield of **CH_2_-Bpy-ZnTPP** (Fig. 2) relative to unsubstituted ZnTPP and found that the emission of **CH_2_-Bpy-ZnTPP** is 15% more intense than that of ZnTPP for samples of equal absorbance at the exciting wavelength. The minor quenching of the bromide complexes demonstrates that heavy atom effects are unimportant and that electron transfer plays a less significant role than in the corresponding picoline complexes. We note also that ZnTPP and zinc tetraphenyl chlorin fluorescence is not quenched by TEOA.^11^

### Photocatalysis

**Fig. 4:**
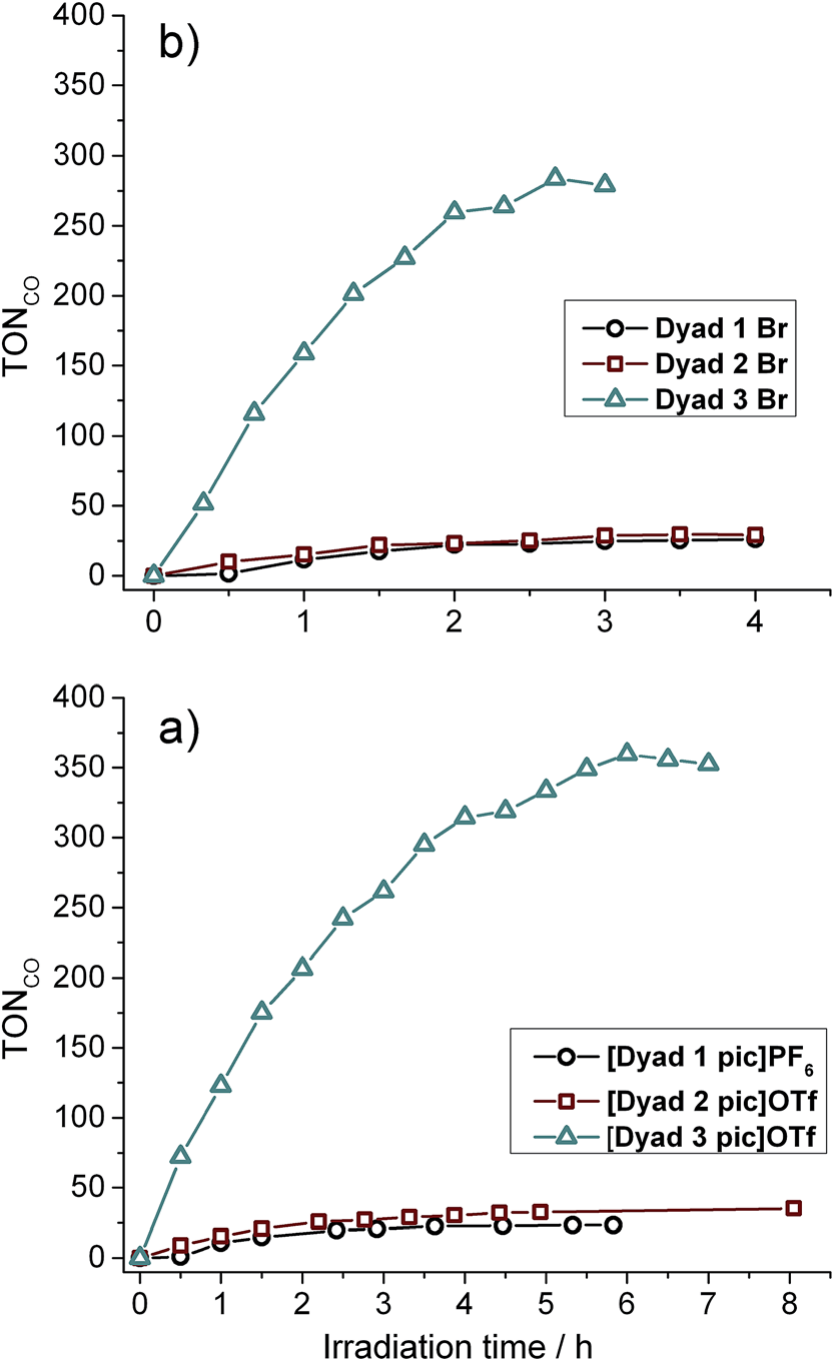
Catalytic activity of all dyads: (a) picoline complexes. (b) bromide complexes. Note the difference in abscissa scales.

All six dyads were tested for CO_2_ photoreduction to CO under irradiation with *λ* > 520 nm in a solution of DMF : TEOA 5 : 1 at 0.05 mM (Fig. 4 and Table 3). Overall turnover frequencies (overall TOF) are calculated over the full period of irradiation, whereas maximum turnover frequencies (max TOF) are calculated over the first hour. The activities of **[Dyad 1 pic]PF_6_** and **[Dyad 2 pic]OTf** have been reported previously^11^ but are included for comparison. **[Dyad 3 pic]OTf** produces CO at a higher rate and is stable over a greater irradiation time and over a greater number of catalytic turnovers than **[Dyad 1 pic]PF_6_** or **[Dyad 2 pic]OTf**. The bromides, **Dyad 1 Br** and **Dyad 2 Br**, show very similar activity to their picoline counterparts. For **[Dyad 2 pic]OTf** and **Dyad 2 Br**, the CO formation plots overlay almost perfectly (Fig. S22†). **Dyad 3 Br** displays a lower TON than **[Dyad 3 pic]OTf** but a higher TOF. The TON of **[Dyad 3 pic]OTf** and **Dyad 3 Br** reach 360 and 270, respectively. We have demonstrated previously that no CO is formed in the absence of TEOA or in the absence of CO_2_. However, two component solutions containing ZnTPP and [ReBpy(CO)_3_(pic)][PF_6_] are active for photocatalysis, reaching a TON of *ca.* 100 in 120 min.^11^ Thus, the activities of **[Dyad 3 pic]OTf** and **Dyad 3 Br** greatly exceed that of the two component system as well as the other dyads.

**Table 3:** Catalytic activities of all dyads in terms of turnover frequencies (TOF_CO_) and turnover numbers (TON_CO_)

Catalyst	Overall TOF_CO_/h^–1^	Maximum TOF/h^–1^	Max TON_CO_^*a*^ ± *σ*
**[Dyad 1 pic]PF_6_**	6	11	27 ± 3
**Dyad 1 Br**	7	12	30 ± 4
**[Dyad 2 pic]OTf**	4	15	32 ± 2
**Dyad 2 Br**	10	15	23^*b*^ ± 6
**[Dyad 3 pic]OTf**	60	123	332 ± 21
**Dyad 3 Br**	95	159	262 ± 19

^*a*^Average of four highest TON_CO_ runs.

^*b*^Average of three highest TON_CO_ runs.

### Photocatalytic intermediates

UV/vis spectra were taken at regular intervals during photocatalysis. We previously reported that significant changes occur in the Q-bands of the porphyrins during CO_2_ photoreduction catalysis by the porphyrin–rhenium dyads.^11^ The Q-bands of porphyrins and their derivatives provide excellent spectroscopic handles in the visible region, providing a clear indication of structural changes. These changes were assigned to formation of chlorin, a reduction product of the porphyrin in which one CC bond of a pyrrole group is saturated.

**Fig. 5:**
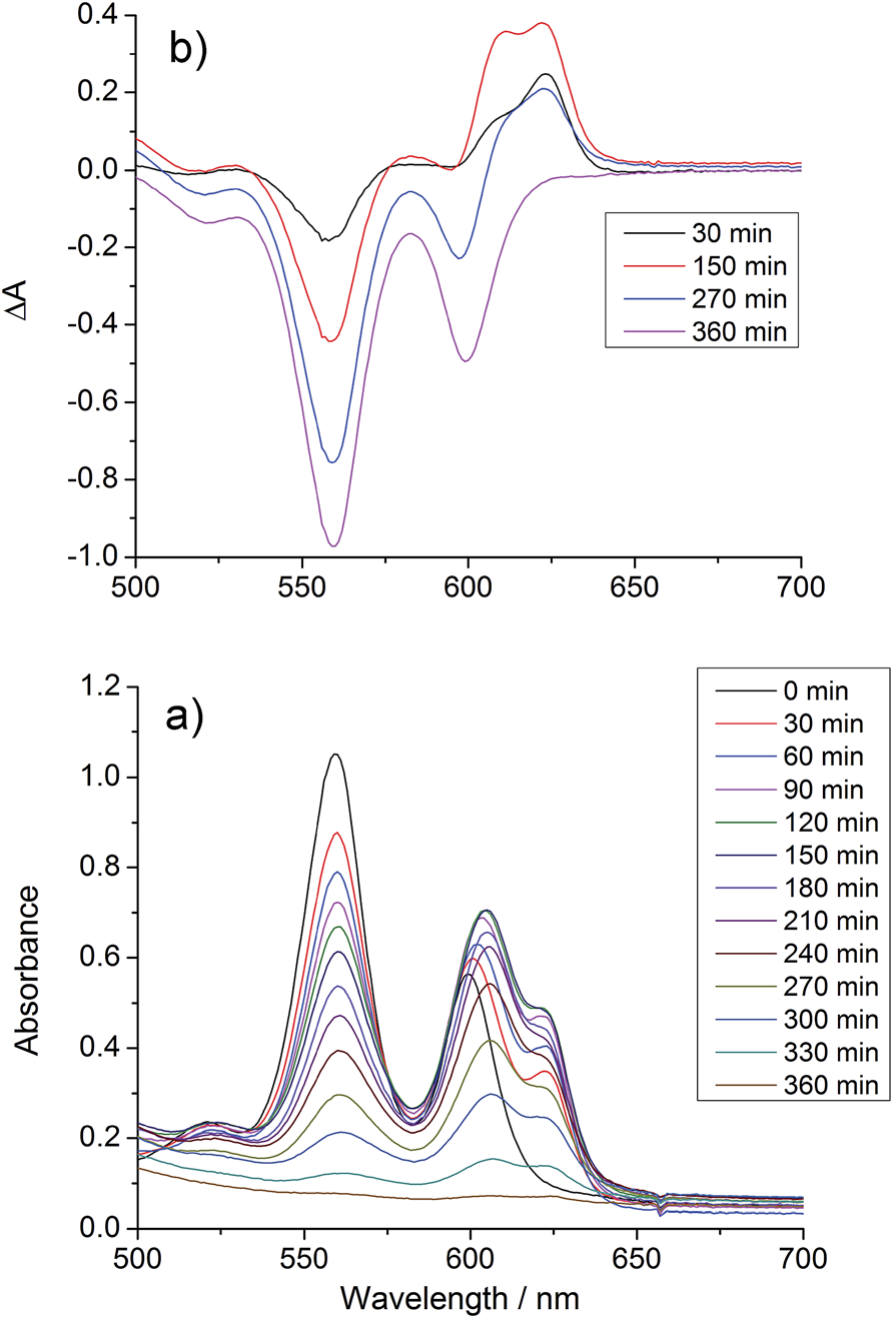
Changes in the UV/vis spectrum of **[Dyad 3 pic]OTf** during CO_2_ photo-reduction: (a) absorption spectrum, (b) difference spectrum, relative to initial spectrum.

The UV/vis spectra of **[Dyad 3 pic]OTf** during catalysis are shown in Fig. 5. At early photolysis times, the Q-bands of the porphyrin decrease in intensity and a product band grows at 625 nm with a shoulder at 610 nm, seen most clearly in the difference spectra (Fig. 5(b)). The relative intensities of the 610 and 625 nm bands change with time. The band at 625 nm may be assigned to zinc chlorin product, while the 610 nm band is assigned to the zinc isobacteriochlorin, the derivative in which two adjacent pyrrole groups are saturated.^94^ This second hydrogenation product is formed in greater amounts for **[Dyad 3 pic]OTf** and persists longer. For all dyads the photocatalytic conditions eventually lead to complete bleaching of the Q-band region of the spectrum.

**Fig. 6:**
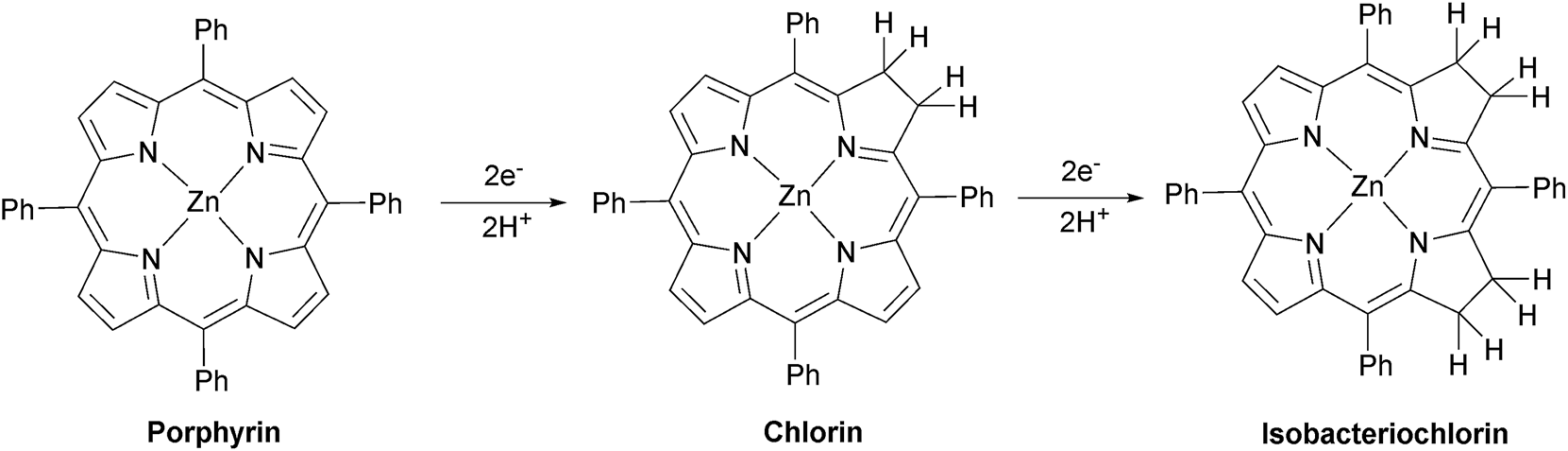
Photo-reduction of zinc porphyrin.

The exact chemical structure of the chlorin cannot be determined from UV/vis spectroscopy alone. It has been shown previously that triethylamine can add to the pyrrole to form both the simple hydrogenation product and a product in which a C–H bond has been formally added across the C=C bond.^95^ A large-scale (50 mg) photolysis (*λ* > 520 nm) was performed on ZnTPP in DMF : TEOA 5 : 1 under Ar and the product was exhaustively extracted into ether after addition of water. The ether was separated and the product dried under vacuum. The ^1^H NMR spectrum of the product dissolved in CDCl_3_ matches the spectrum of an authentic sample of zinc tetraphenylchlorin (Fig. S23†), demonstrating that the major product is formed by simple hydrogenation (Fig. 6).

ESI-mass spectrometry measurements were made on samples from CO_2_ photoreduction by [Re(Bpy)(CO)_3_(pic)][PF_6_] and zinc tetraphenyl porphyrin (ZnTPP). Zinc possesses several isotopes of significant abundance producing a pattern that spans several *m*/*z* units. As a result, the signals for the various hydrogenation products of zinc porphyrin overlap closely. The signals obtained centre around *m*/*z* = 680 and match well with the calculated isotope pattern for a mixture of ZnTPP and the di-hydrogenated (chlorin) and tetra-hydrogenated (isobacteriochlorin) products (Fig. S24† and 6).

### Substitution in **[Dyad 2 pic]OTf** and **Dyad 2 Br**

**Fig. 7:**
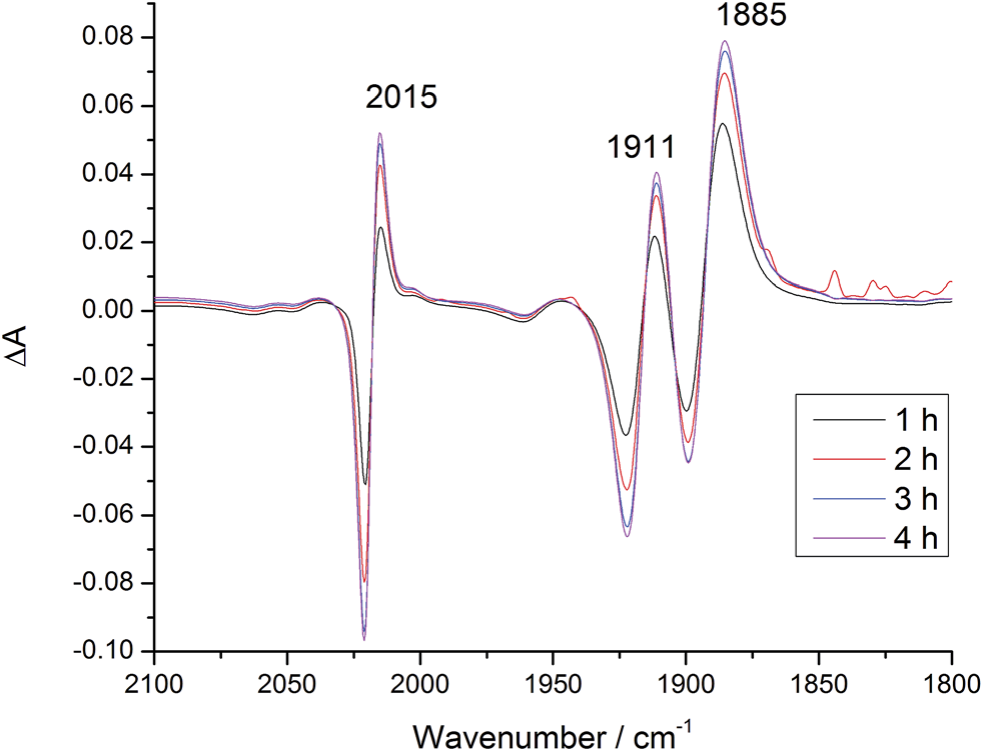
IR difference spectra of **Dyad 2 Br** in DMF after addition of TEOA (DMF : TEOA 5 : 1), under CO_2_ and with *λ* > 520 nm irradiation. Difference spectra relative to before addition of TEOA and CO_2_.

Recently Ishitani and co-workers reported the thermal substitution of [Re(Bpy)(CH_3_CN)(CO)_3_][PF_6_] in DMF and DMF : TEOA 5 : 1 mixtures to produce [Re(Bpy)(CO)_3_DMF]^+^ and Re(OCH_2_CH_2_NR_2_)(Bpy)(CO)_3_ (R = CH_2_CH_2_OH).^53^ This observation could have significant implications for the energetics and photochemistry of the dyad bromide complexes in which the charge at rhenium would change. **[Dyad 2 pic]OTf** and **Dyad 2 Br** were investigated for thermal substitution in DMF or DMF : TEOA 5 : 1 by monitoring with IR spectroscopy. In DMF neither **[Dyad 2 pic]OTf** nor **Dyad 2 Br** showed substitution over 5 h (Fig. S25 and S26†). This finding contrasts with the report on [Re(Bpy)(CH_3_CN)(CO)_3_][PF_6_] in which [Re(Bpy)(CO)_3_DMF]^+^ is observed.^53^ However, CH_3_CN is probably more labile than bromide or 3-picoline. Addition of TEOA (giving DMF : TEOA 5 : 1) to **[Dyad 2 pic]OTf** led to the gradual appearance of signals (12% conversion in 30 min) at 2006, 1895 and 1881 cm^–1^ (Fig. S27†).^96^ A similar experiment with **Dyad 2 Br** produced no change thermally, but a product with bands at the same wavenumbers appeared on photolysis with *λ* > 520 nm under N_2_ (Fig. S28†). In an attempt to increase conversion and the signal of the substitution product, CO_2_ was bubbled through **Dyad 2 Br** in DMF : TEOA 5 : 1 under *λ* > 520 nm irradiation. This time, different signals were observed at 2015, 1911 and 1885 cm^–1^ that correspond closely to those reported^53^ for the carbonato complex (Fig. 7 and S28†).

Considering the excellent fit to Ishitani's data for Re(OCH_2_CH_2_NR_2_)(Bpy)(CO)_3_, the product from **[Dyad 2 pic]OTf** may be assigned as **Dyad 2 OCH_2_CH_2_NR_2_**. We are not able to show definitively whether the product from **Dyad 2 Br** is the same or the analogue where the porphyrin has been reduced to chlorin, since the timescale for hydrogenation is similar to the timescale for reaction with TEOA. Nevertheless, the IR evidence supports CO_2_ insertion into the metal–oxygen bond to form species containing the Re{OC(O)OCH_2_CH_2_NR_2_}(Bpy)(CO)_3_ unit.^53^

### Picosecond time-resolved infrared spectroscopy – picoline complexes

**Fig. 8:**
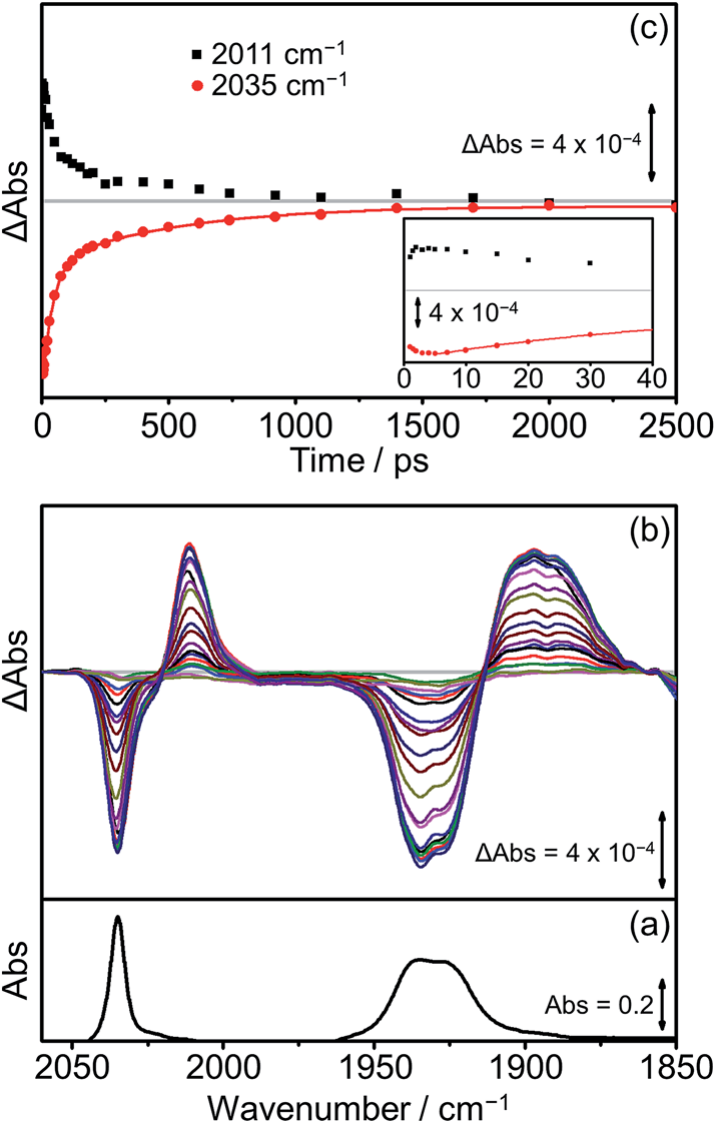
FTIR and TRIR spectra of **[Dyad 2 pic]OTf** in CH_2_Cl_2_: (a) FTIR ground state spectrum; (b) TRIR difference spectra taken between 1 and 2500 ps after flash photolysis at 560 nm; (c) TRIR single point kinetic traces for the formation and decay of the CS product (black squares, 2011 cm^–1^) and the depletion and reformation of the ground state bands (red dots, 2035 cm^–1^). The solid red line is a bi-exponential fit of the data. Inset shows expansion of the first 40 ps.

Extensive previous TRIR investigations of the excited states of Re(Bpy)(CO)_3_ derivatives^34^,^68^,^80^ show that formation of ^3^MLCT excited states result in high frequency shifts of the carbonyl vibrations, whereas charge transfer to the Re(CO)_3_ results in substantial low frequency shifts.^34^ The photophysics and photochemistry of **[Dyad 1 pic]OTf** have previously been investigated using TRIR spectroscopy in PrCN.^34^ Excitation of **[Dyad 1 pic]OTf** at 600 nm resulted in the initial formation of an excited state localised on the porphyrin moiety of the dyad, followed by subsequent electron transfer to the Re(diimine) ligand generating a charge-separated (CS) state. The CS state reached a maximum within 10 ps and decayed over 40 ps. Charge recombination back to the porphyrin moiety *via* a hot ground (HG) state regenerated the parent complex within 200 ps. In addition, a sharp peak in the TRIR spectra at 2026 cm^–1^ could be observed during the first 5 ps, which was tentatively assigned to the formation of an intraligand (IL) ππ* excited state.^97^ The complexity of the transient spectroscopy was reconciled with a model which postulates that the dyad molecules adopt a range of conformations each with their own kinetics. We have performed an analogous set of TRIR experiments on **[Dyad 2 pic]OTf** and **[Dyad 3 pic]OTf** following excitation into the Q(1, 0) band at 560 nm in CH_2_Cl_2_. For both **[Dyad 2 pic]OTf** and **[Dyad 3 pic]OTf**, only the CS state could be detected after excitation and neither the formation of a ππ* excited state nor HG state were observed at any time delays. The TRIR spectra recorded between 1 and 2500 ps following flash photolysis of **[Dyad 2 pic]OTf** are shown in Fig. 8(b). Two negative signals, one sharp at 2035 cm^–1^ and one broad at 1931 cm^–1^ can be observed, corresponding to bleaching of the ground state carbonyl bands on the Re moiety. Transient *ν*(CO) bands can also be observed in the TRIR spectra with a sharp feature at 2011 cm^–1^ and one broad absorbance at 1895 cm^–1^. The positions of these transient peaks correspond closely to those of the CS state obtained following the excitation of **[Dyad 1 pic]OTf** in PrCN (2007 and *ca.* 1896 cm^–1^).^34^Fig. 8(c) shows the kinetics of the CS species and the parent bleach. The CS species is formed with a risetime of *ca.* 2 ps and decays over the subsequent 2.5 ns back to the ground state complex (Fig. 8(c), black squares). The regrowth of the parent bleach at 2035 cm^–1^ was fitted to a bi-exponential function with lifetimes of 42 ± 2 and 515 ± 35 ps (Fig. 8(c), red dots) (proportions *ca.* 68% and 32%, respectively). Since increasing dilution of the solution from 1.5 mM to 1.0 mM had little effect on the kinetics, we suggest that the bi-exponential decay is not due to dimer formation, as observed in the X-ray diffraction experiments, but more likely due to presence of two conformers present with different lifetimes.

**Fig. 9:**
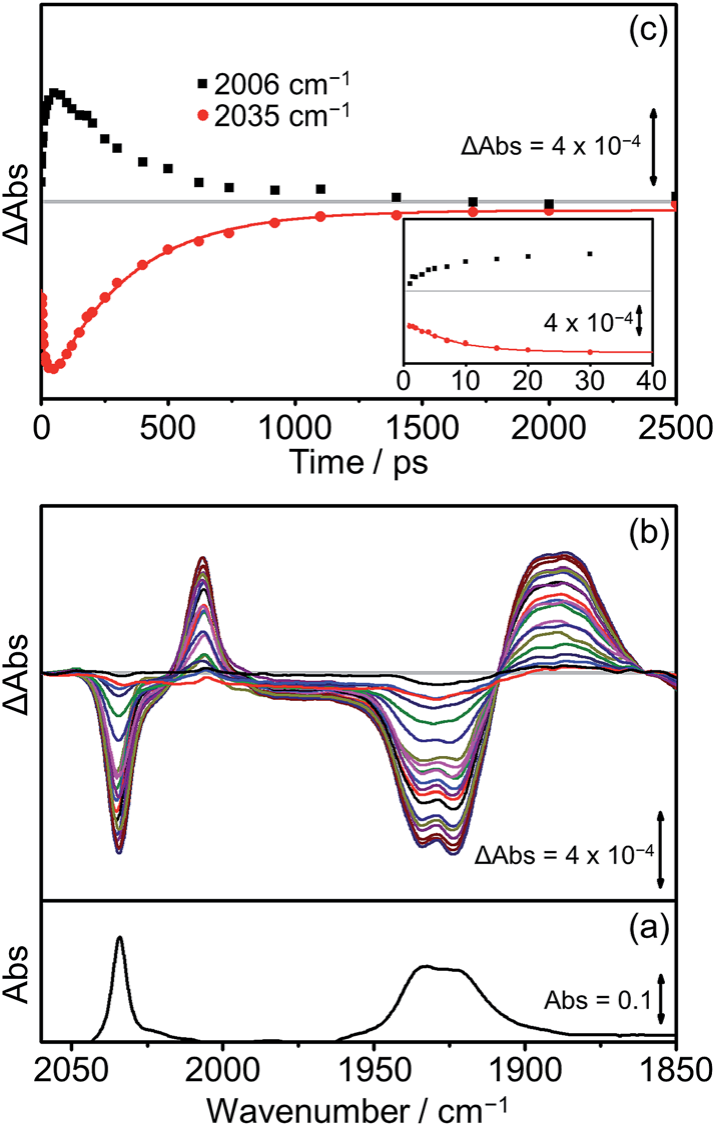
FTIR and TRIR spectra of **[Dyad 3 pic]OTf** in CH_2_Cl_2_: (a) FTIR ground state spectrum; (b) TRIR difference spectra taken between 1 and 2500 ps after flash photolysis at 560 nm; (c) TRIR single point kinetic traces for the formation and decay of the CS product (black squares, 2006 cm^–1^) and the depletion and reformation of the ground state bands (red dots, 2035 cm^–1^). The solid red lines are mono-exponential fits of the data. Inset shows expansion of the first 40 ps.

The TRIR spectra of **[Dyad 3 pic]OTf** obtained following excitation (Fig. 9(b)) show qualitatively similar band positions and processes to those obtained for **[Dyad 2 pic]OTf**. Bleaching of the ground state can be observed (2035 cm^–1^ and 1930 cm^–1^) as well as the formation of two transient peaks (2006 and 1890 cm^–1^) corresponding to the appearance of the CS species. However, in the case of **[Dyad 3 pic]OTf** the growth and decay of the CS state is different to that of **[Dyad 2 pic]OTf**. The CS species (Fig. 9(c), black dots) of **[Dyad 3 pic]OTf** grows in with a lifetime of 8 (±1) ps and depletes following a mono exponential decay with lifetime of 320 (±15) ps. The kinetics of the parent bleach closely match those of the CS species, indicating that the transient decays directly back to the ground state. The kinetic lifetimes for the formation and decay of the CS species in **[Dyad 1 pic]OTf**, **[Dyad 2 pic]OTf** and **[Dyad 3 pic]OTf** and the down-frequency shifts of the *ν*(CO) bands are summarized in Table 4.

**Table 4:** Lifetimes for the growth and decay of the CS state

Dyad (solvent)	Risetime (ps)	Lifetime (ps)	Δ*ν* of *ν*(CO)_sym_ of CS state (cm^–1^)
**[Dyad 1 pic]OTf** (PrCN)^34^	<1	40 ± 4	–24
**[Dyad 2 pic]OTf** (CH_2_Cl_2_)	*ca.* 2	42 ± 2 (and 515 ± 35)	–24
**[Dyad 3 pic]OTf** (CH_2_Cl_2_)	8 ± 1	320 ± 15	–29

### Picosecond time-resolved infrared spectroscopy – bromide complexes

The photophysics and photochemistry of **Dyad 1 Br**, **Dyad 2 Br** and **Dyad 3 Br** were monitored using TRIR spectroscopy following excitation at 560 nm in THF. All TRIR spectra were obtained in THF since the dyads are not sufficiently soluble in CH_2_Cl_2_. In general, the TRIR spectra of the bromide dyads are more complex than those of the picoline dyads, with multiple transient species observable in the spectra.

**Fig. 10:**
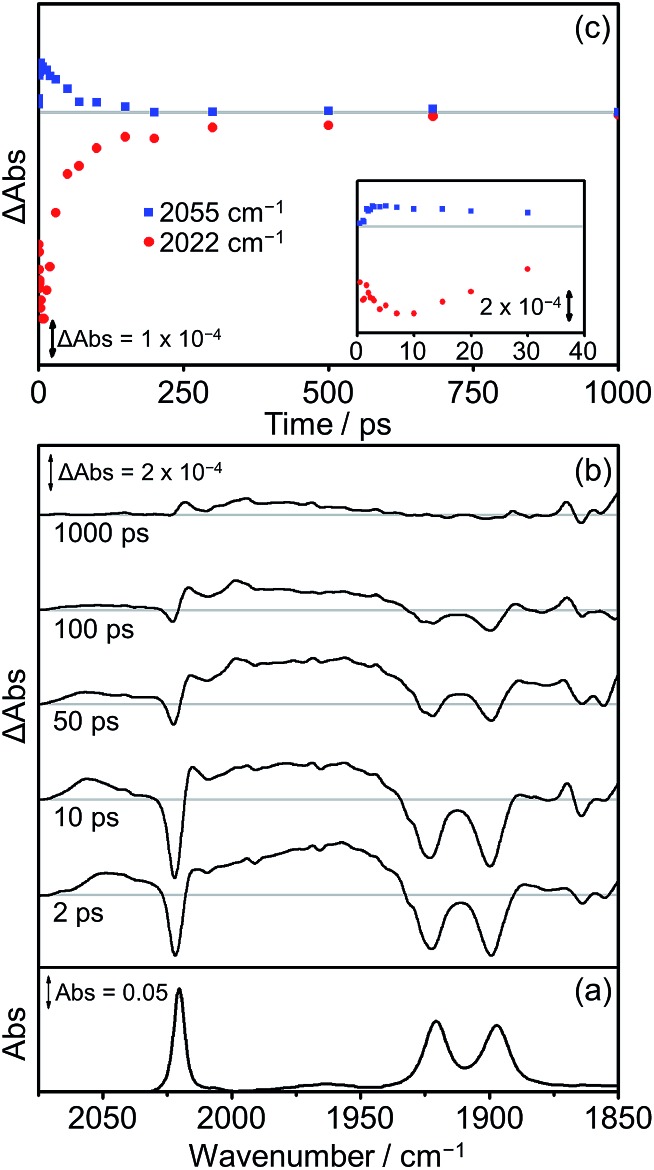
FTIR and TRIR spectra of **Dyad 1 Br** in THF: (a) FTIR ground state spectrum; (b) TRIR difference spectra taken at 2, 10, 50, 100 and 1000 ps after flash photolysis at 560 nm; (c) TRIR single point kinetic traces for the depletion and reformation of the ground state bands (red dots, 2022 cm^–1^) and the growth and decay of the ^3^MLCT excited state (blue squares, 2055 cm^–1^). Inset shows expansion of the first 40 ps.

The TRIR spectra obtained following excitation of **Dyad 1 Br** are shown in Fig. 10. Three negative bands are observed corresponding to the parent complex at 2022, 1922 and 1900 cm^–1^. At early time delays (<50 ps) a band at 2055 cm^–1^ and a broad band at *ca.* 1960 cm^–1^ can be observed, characteristic of the high frequency shift associated with the formation of a ^3^MLCT state on the Re moiety of the dyad.^68^,^77^,^98^,^99^ This ^3^MLCT excited state is formed initially <10 ps after excitation from vibrationally hot excited states and decays over the subsequent 250 ps (Fig. 10(c), blue squares). The formation of peaks at 1998 cm^–1^ and 2015 cm^–1^ can also be observed on a similar timescale to the decay of the ^3^MLCT state. The peak at 1998 cm^–1^ is analogous to observations made on the picoline dyads (see above) and is assigned to the formation of a CS state. The corresponding lower energy bands associated with the CS species can be observed at *ca.* 1880 cm^–1^, but due to their weak intensity, the exact band positions could not be determined. The band at 2015 cm^–1^ suggests the simultaneous formation of an IL ππ* excited state,^77^,^97^ similar to that observed following the photolysis of **[Dyad 1 pic]OTf** in PrCN.^34^ The associated low energy bands of the IL ππ* excited state cannot be observed as they are low intensity and fall in a similar region of the spectrum to the ground state bleach. Bleaching of the ground state does not reach a maximum negative signal until 15 ps, and it recovers over the subsequent 1000 ps (Fig. 10(c), red dots). The recovery of the signal at 2022 cm^–1^ occurs over two distinct timescales. The first (0–100 ps) is mainly associated with deactivation of the ^3^MLCT and the second (100–1000 ps) is principally due to the decay of the CS and ππ* excited state. The kinetics of the CS state and the IL ππ* excited state were not fully determined as the bands are weak and overlap with other bands in this region of the spectrum.

**Fig. 11:**
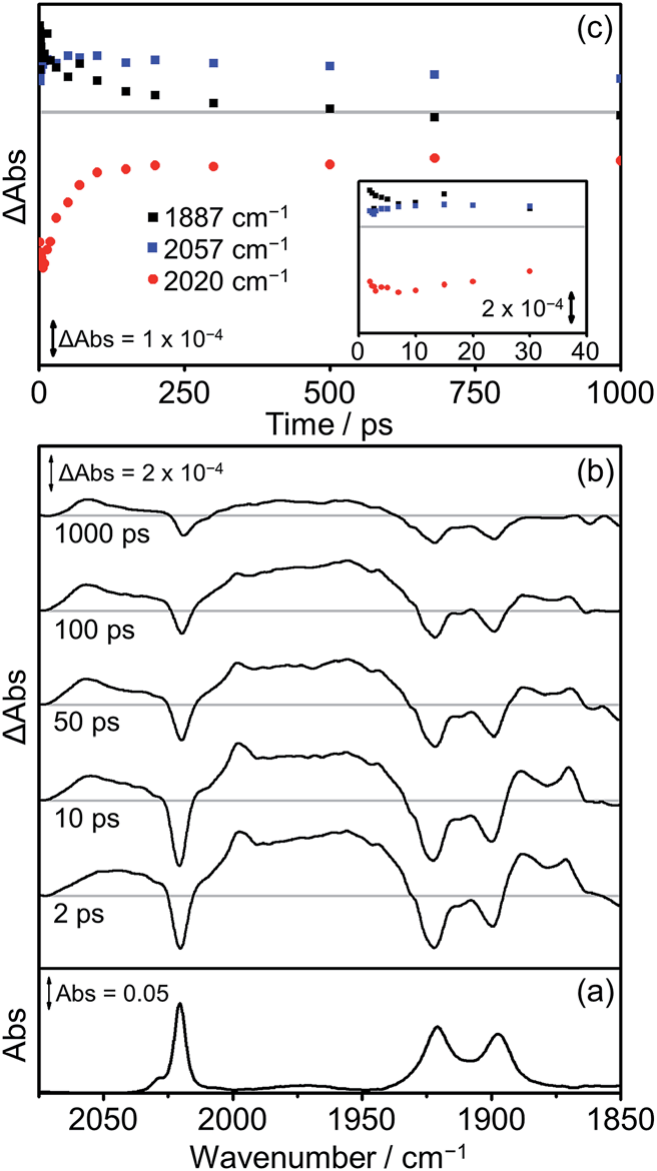
FTIR and TRIR spectra of **Dyad 2 Br** in THF: (a) FTIR ground state spectrum; (b) TRIR difference spectra taken between 2, 10, 50, 100 and 1000 ps after flash photolysis at 560 nm; (c) TRIR single point kinetic traces for the CS product (black squares, 1887 cm^–1^), the ground state bands (red dots, 2020 cm^–1^) and the ^3^MLCT excited state (blue squares, 2057 cm^–1^). Inset shows expansion of the first 40 ps.

The TRIR spectra obtained following excitation of **Dyad 2 Br** are shown in Fig. 11. Parent bleaches at 2020, 1922 and 1900 cm^–1^ can be observed as well as the formation of two transient species (Fig. 11(b)). At all time delays, bands at 2057 cm^–1^ and *ca.* 1975 cm^–1^ (broad) are visible, associated with the formation of a ^3^MLCT excited state on the Re moiety. This ^3^MLCT state is initially formed from vibrationally hot excited states at time delays <10 ps. In addition, bands at 1997, 1887 and 1871 cm^–1^ can be observed <500 ps after excitation, which are assigned to the formation of a CS state. The CS species grows in on a timescale faster than 2 ps and decays over the subsequent 1000 ps (Fig. 11(c), black squares) as the parent bleach partially recovers (65%, Fig. 11(c), red dots). An IL ππ* excited state was not observed at any time delay in this experiment. At 500 ps after photolysis, the only bands visible in the TRIR spectrum are those originating from the ^3^MLCT and these bands along with the parent bleaches do not change intensity significantly on the timescale of this experiment (up to 1000 ps).

**Fig. 12:**
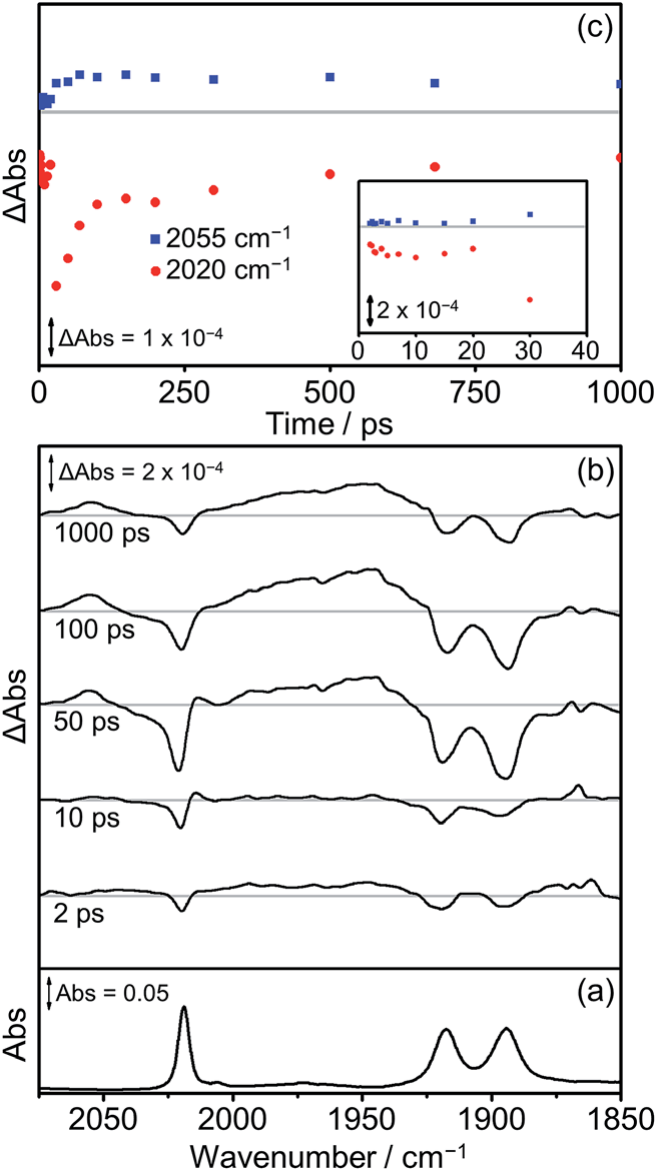
FTIR and TRIR spectra of **Dyad 3 Br** in THF: (a) FTIR ground state spectrum; (b) TRIR difference spectra taken 2, 10, 50, 100 and 1000 ps after flash photolysis at 560 nm; (c) TRIR single point kinetic traces for the ground state bands (red dots, 2020 cm^–1^) and the ^3^MLCT excited state (blue squares, 2055 cm^–1^). Inset shows expansion of the first 40 ps.

The TRIR spectra recorded after flash photolysis of **Dyad 3 Br** are shown in Fig. 12. Bands associated with the formation of a ^3^MLCT excited state at 2055 cm^–1^ and *ca.* 1975 cm^–1^ (broad) grow in over the first 100 ps and do not deplete significantly up to 1000 ps after excitation. In addition, an IL ππ* excited state band at 2014 cm^–1^ can be observed that grows in over the first 30 ps and completely decays by 100 ps. The low energy bands of the IL ππ* excited state cannot be observed as they are weak in intensity and overlap with the ground state bleaches. The ^3^MLCT state is probably formed *via* energy transfer from the porphyrin ππ* excited state.^97^ This is energetically feasible as the higher energy emission maximum of **Dyad 1 Br** is at 606 nm, compared to the emission maximum for the ^3^MLCT state of ReBr(Bpy)(CO)_3_ at 620 nm.^81^ In contrast to **Dyad 1 Br** and **Dyad 2 Br**, a CS state was not observed following the photolysis of **Dyad 3 Br**. The ground state bleach reaches a maximum at 30 ps and has recovered by 65% at 1000 ps after excitation. Through a separate ns-TRIR experiment we determined that the ^3^MLCT state decays with a lifetime of *ca.* 2 ns as the parent complex reforms. However, this experiment had to utilise a 532 nm excitation pulse which is not ideal as it falls at the edge of the porphyrin Q band absorption and led to relatively weak TRIR signals. We examined the possible quenching of the ^3^MLCT excited state with the addition of TEOA to the solution of **Dyad 3 Br**. Reductive quenching of the ^3^MLCT state is expected to be a small component of the decay because of the short excited state lifetime. However, no reductive quenching was observed. Given the low signal-to-noise of these measurements due to the unfavourable excitation wavelength, we can only state that if quenching occurs then it represents less than 1% of the ^3^MLCT decay.

## Discussion

### Energetics of electron transfer

The change in free energy for the dyad picoline cations on intramolecular electron transfer from the excited state of the sensitizer to rhenium can be estimated using eqn (1) where *E*_ox_ and *E*_red_ are taken as the potentials for the first oxidation of the sensitizer and first reduction of the rhenium, respectively. The potentials were estimated from cyclic voltammograms measured in CH_2_Cl_2_. For *E*_00_, we used the highest energy emission maximum of the sensitizer, measured at room temperature. The potentials, emission maxima, driving forces and maximum TON_CO_ are given in Table 5. We do not report quantum yields for CO production because there is significant photoreaction at the porphyrin during catalysis.

1 ΔG*ox = *E*_ox_ – *E*_red_ – *E*_00_

**Table 5:** Lowest energy emission maxima and driving force of electron transfer excluding the electrostatic term for the catalytic systems tested, (all in CH_2_Cl_2_, for half-wave potentials see Table 1)

Dyad	*E*_00_/eV	ΔG*ox/eV	Max TON_CO_ ± *σ*
**[Dyad 1 pic]PF_6_**	2.07	–0.35	27 ± 3
**Dyad 1 Br**	2.07	–0.08	30 ± 4
**[Dyad 2 pic]OTf**	2.06	–0.32	32 ± 2
**Dyad 2 Br**	2.07	–0.17	23 ± 6
**[Dyad 3 pic]OTf**	2.07	–0.06	332 ± 21
**Dyad 3 Br**	2.08	0.12	262 ± 19

For the bromide dyads, we expect an additional electrostatic contribution to the free energy of electron transfer, since the electron transfer generates a pair of charges. The edge-to-edge distance from porphyrin to Bpy may be regarded as the minimum distance for electron transfer and is measured at 8.0 Å in the crystal structure of **[Dyad 1 pic]PF_6_**. The electrostatic contribution in CH_2_Cl_2_ is calculated as –0.20 eV and may be taken as an upper limiting value for **Dyad 1 Br**. The corresponding value for **Dyad 2 Br** would be significantly less negative, while that for **Dyad 3 Br** may be more negative at *ca.* –0.27 eV because of its ability to fold about the CH_2_ group. However, in DMF, the solvent used for CO_2_ reduction, these values become of little importance because of the high dielectric constant of the solvent: –0.04 eV for **Dyad 1 Br** and –0.05 eV for **Dyad 3 Br**. Table 5 lists the data for the bromide complexes without the electrostatic contributions.

The values of ΔG*ox for electron transfer are negative for **[Dyad 1 pic]PF_6_** and **[Dyad 2 pic]OTf**. The values of ΔG*ox in Table 5 for **[Dyad 3 pic]OTf** and **Dyad 1 Br** are close to zero, while that for **Dyad 3 Br** is positive. The bromide dyads have completely different potentials from the picoline dyads yet their photocatalytic behaviour is very similar and sometimes superimposable. Furthermore, **Dyad 3 Br** is very active, yet the driving force in DMF is not favourable for electron transfer. Considering just the picoline complexes, the greater is the driving force for electron transfer, the lower is the observed maximum turnover number. We can deduce from these points that the porphyrin dyad bromides are not the active species in photocatalysis. However, we have previously shown that the driving force for electron transfer from the excited state of zinc tetraphenylchlorin to Re complexes is 150 meV more negative than that for the excited state of zinc tetraphenylporphyrin.^11^ Thus, it is possible that bromide dyads become more active on reduction to the chlorin derivative (see Mechanism section of Discussion, below). The corresponding values of the reduction potentials of Re(OCH_2_CH_2_NR_2_)(Bpy)(CO)_3_ (R = CH_2_CH_2_OH) and related dyads are not known, but we would expect them to be close to those of the bromide complexes. The reduction potential of Re(OCOOCH_2_CH_2_NR_2_)(Bpy)(CO)_3_ (R = CH_2_CH_2_OH) is reported to be very similar to that of the simple bromide complex.^53^

### Emission spectroscopy

Emission data allow us to derive quantum yields for fluorescence from the singlet ππ* state and estimates of the intramolecular quenching rate (Table 2). The quantum yields are based on the quantum yield of fluorescence for ZnTPP in toluene of 3%.^93^ Across both the picoline and bromide dyads, steady state emission quantum yields and emission lifetimes increase significantly as photocatalytic activity increases. This lack of correlation may be resolved if some of the dyads are emissive but inactive with respect to charge separation, while others are non-emissive but undergo charge separation. Thus the fluorescence data show that there are significant excited state populations that do not directly lead to photocatalysis. This diversity of behaviour is attributed to conformers which are not predisposed to the required electron transfer process. If there was no issue of multiple conformers, all the molecules would end up in the state with the shortest rise-time following formation of the porphyrin π–π* S_1_ state.

### Time resolved infrared spectroscopy

There are some striking differences in the TRIR spectra and kinetics obtained between the different dyads. The TRIR spectra of the three picoline derivatives are dominated by formation of the CS state. Neither **[Dyad 2 pic]OTf** nor **[Dyad 3 pic]OTf** form ππ* excited states (in CH_2_Cl_2_) detectable by TRIR spectroscopy unlike **[Dyad 1 pic]OTf** (in PrCN). Since the risetimes of the CS state are in the range 1–10 ps, whereas the values of *k*_Q_^–1^ derived from emission data are in the range 100–17 000 ps, we conclude that the CS states and the emissive ππ* states on the porphyrin arise from different conformers of the picoline complexes. The crystal structure shows that the torsional angles for **[Dyad 1 pic]PF_6_** are not ideal for electron transfer and represent one conformer out of many that may be present in solution (Fig. 3). Comparison shows that **[Dyad 3 pic]OTf** exhibits the longest risetime for charge separation and the longest lived charge-separated state (Table 4). Only **[Dyad 1 pic]OTf** undergoes charge recombination *via* a hot ground state.^34^,^100^ Although the lifetimes of the CS states correlate with photoactivity, they are extremely short if bimolecular reaction is to occur with any species other than either triethanolamine or DMF which are components of the solvent, even for **[Dyad 3 pic]OTf**.

The TRIR spectra of the bromide complexes are very different from those of the picoline complexes. In the bromide complexes, we observe a ^3^MLCT state in all three dyads and the CS state can only be clearly observed in **Dyad 1 Br** and **Dyad 2 Br**. These observations are consistent with the driving force calculations above. The ^3^MLCT states of **Dyad 2 Br** and **Dyad 3 Br** have lifetimes on the ns timescale. The risetimes of the ^3^MLCT states are in the range of tens of picoseconds which is again incompatible with the rate of quenching of the ππ* states. We suggest that these differences reflect the presence of multiple conformers. The absence of the CS state of **Dyad 3 Br** appears remarkable considering its strong photocatalytic activity.

### Mechanism

On photo-excitation, **[Dyad 2 pic]OTf** and **[Dyad 3 pic]OTf** form charge-separated states, as seen for **[Dyad 1 pic]OTf**.^34^ The close match in the photocatalysis curves for **[Dyad 2 pic]OTf** and **Dyad 2 Br** (Fig. S22†) suggests that these compounds share a catalytically active species. The other two pairs also exhibit strong similarity in the curves. The parallel photocatalytic behaviour contrasts with the very different excited states observed by TRIR spectroscopy (see above). We have presented evidence from steady-state spectroscopy that the photocatalysts undergo reaction with triethanolamine both at the porphyrin centre and the rhenium centre. Photoreaction at the porphyrin causes initial 2-electron hydrogenation to the chlorin and subsequently a further 2-electron hydrogenation to the isobacteriochlorin. Thermal reaction of **[Dyad 2 pic]OTf** and photoreaction of **Dyad 1 Br** yield evidence for formation of Re(OCH_2_CH_2_NR_2_)(Bpy)(CO)_3_ derivatives. In addition, triethanolamine and DMF are capable of coordinating to zinc, probably forming equilibrium mixtures. An electron can be supplied to the oxidised porphyrin by the fifth ligand on zinc. Taken together with the arguments presented above on energetics and TRIR spectra, these considerations indicate that the dyads act as pre-catalysts. Within the first 30 min of irradiation, significant reduction to chlorin and reaction with triethanolamine occurs, probably generating the true photocatalysts.

## Conclusions

We have shown that a new design of zinc porphyrin–Re bipyridine tricarbonyl dyad with a methylene spacer is active for the photocatalytic reduction of CO_2_ to CO with *λ* > 520 nm irradiation. The TON is higher than those for previously reported dyads by a factor of ten and there is a major increase in TOF. These figures exceed those for the two component system ZnTPP + [ReBpy(CO)_3_(pic)][PF_6_] by a factor of three.^11^ The benefit of using a saturated bridge between Bpy and the porphyrin is in accord with Ishitani's binuclear Ru–Re complexes which were also most effective with a saturated bridge.^10^ The most likely reason for the enhanced activity is the flexibility for the Re(Bpy) unit to adopt the best orientation and closest approach to the porphyrin unit. Furthermore, in these dyads the groups at the 4 and 4′ positions of the Bpy are CH_3_ and CH_2_R, making them electronically similar to simple Bpy and dimethylbipyridine. The mononuclear Re complexes of Bpy and dimethylbipyridine are some of the most active electro- and photo-catalysts. **Dyad 1 Br** and **Dyad 2 Br** show very similar photocatalytic behaviour to their picoline analogues. **Dyad 3 Br** is slightly less effective in TON, but slightly more effective in TOF than **[Dyad 3 pic]OTf**. Emission quenching of the rhenium complexes relative to the rhenium-free zinc porphyrins can be observed in all three picoline dyads and in **Dyad 1 Br** and **Dyad 2 Br**. The amount of quenching decreases in the order **Dyad 1** > **Dyad 2** > **Dyad 3** for each of the picoline and bromide series, with minimal quenching for **Dyad 3 Br**.

The dyads undergo photoreduction resulting in hydrogenation of the porphyrin at one pyrrole ring to give a chlorin species, followed by formation of an isobacteriochlorin and eventually complete bleaching. Previous results indicate that the chlorin intermediates are active catalysts.^11^ It is likely that the isobacteriochlorin intermediates are active also. Complete bleaching renders the dyads unable to absorb visible light and is one route for deactivation. The dyads react with triethanolamine in DMF to form alkoxide complexes containing a Re(OCH_2_CH_2_NR_2_)Bpy(CO)_3_ moiety which undergoes CO_2_ insertion. The picoline complexes undergo this transformation thermally while the bromide complexes require irradiation.

**[Dyad 3 pic]OTf** undergoes charge separation in 8 ps and the charge-separated state has a lifetime of 320 ps. For comparison, **[Dyad 2 pic]OTf** undergoes faster charge separation but the majority of the CS photoproduct decays much faster (with time constant of 42 ps). The bridge in **Dyad 3** has slowed down charge separation and charge recombination. The charge-separated state is one order of magnitude longer-lived in **Dyad 3** than in **Dyad 2**. The bromide complexes show very different photochemical behaviour on the ps timescale with combinations of either ^3^MLCT and CS, or ^3^MLCT and IL excited state products. **Dyad 1 Br** and **Dyad 2 Br** form some CS product, whereas **Dyad 3 Br** does not. The CS state is unlikely to be responsible for the activity of the bromide complexes since **Dyad 3 Br** is very active. All three bromide dyads display formation of a ^3^MLCT state, the lifetimes of which decrease in the order **Dyad 3 Br** > **Dyad 2 Br** > **Dyad 1 Br**, in line with their photocatalytic activities. The ^3^MLCT state may be responsible for the activity of the bromide dyads. As expected for such short lifetimes, ns-TRIR experiments on **Dyad 3 Br** showed little or no bimolecular reaction with TEOA but this cannot be ruled out. Thus the bromide complexes display very similar photocatalytic behaviour to the picoline complexes but totally different excited states.

Taken together, the data strongly suggest that the active photocatalyst is formed by a combination of reaction of triethanolamine at rhenium and photoreduction of the porphyrin. We have previously shown that zinc chlorin is more reducing than zinc porphyrin^11^ and may allow for the formation of significant amounts of charge-separated state in the bromide complexes as well as the picoline complexes. This hydrogenation can also explain why the picoline dyads are not de-activated on thermal substitution of picoline for the anionic alkoxide/carbonato complexes, which would be expected to have similar reduction potentials to the bromides. TRIR experiments demonstrate that bimolecular reaction of the ^3^MLCT state of **Dyad 3 Br** is minimal and thus support the chlorin theory.

## Supplementary Material

Supplementary information

Crystal structure data
