# Supplementary material for: Comparison of rhenium–porphyrin dyads for CO2 photoreduction: photocatalytic studies and charge separation dynamics studied by time-resolved IR spectroscopy
Source: Chem Sci. 2015 Aug 20;6(12):6847–64. doi: 10.1039/c5sc02099a (PMC5947513; doi:10.1039/c5sc02099a)
Supplement: Supplementary file 1 [file SC-006-C5SC02099A-s001.pdf]

Electronic Supplementary Material (ESI) for Chemical Science.
This journal is © The Royal Society of Chemistry 2015

Supporting information for:

Comparison of rhenium-porphyrin dyads for CO_2 photoreduction: photocatalytic studies and charge separation dynamics studied by time-resolved IR spectroscopy

Christopher D. Windle,[a] Michael W. George,[b,c*] Robin N. Perutz,[a*] Peter A. Summers,[b,c] Xue Zhong Sun,[b] and Adrian C. Whitwood[a]

(a) Department of Chemistry, University of York, Heslington, York, YO10 5DD, U.K.
(b) School of Chemistry, University of Nottingham, Nottingham, NG7 2RD, U.K.
(c) Department of Chemical and Environmental Engineering, The University of Nottingham Ningbo China, Ningbo, 315100, China

*Corresponding author. Email: robin.perutz@york.ac.uk, mike.george@nottingham.edu.cn

Primary data will be made available at DOI: 10.15124/4335e576-3737-4968-841b-076f8a4658ca

Table of contents

NMR spectra and assignments S1

Mass spectrometry of **[Dyad 3 pic]OTf** S8

Cyclic voltammetry S9

Crystallographic data S10

Emission spectra S13

TON plot for **[Dyad 2 pic]OTf** and **Dyad 2 Br** S15

Characterisation of hydrogenated porphyrin S16

IR spectra of ligand substitution S17

Dynamics in UV/vis spectra during catalysis S20

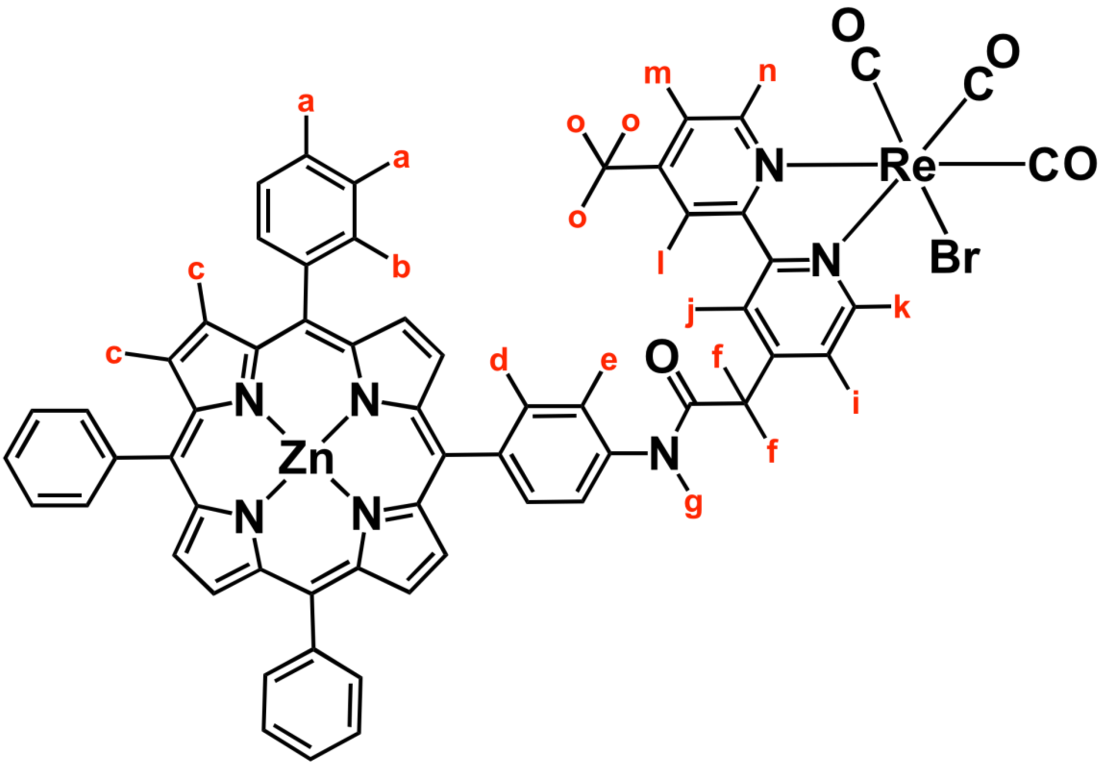

Figure S 1. ^{1}H NMR assignment of **Dyad 3 Br**.

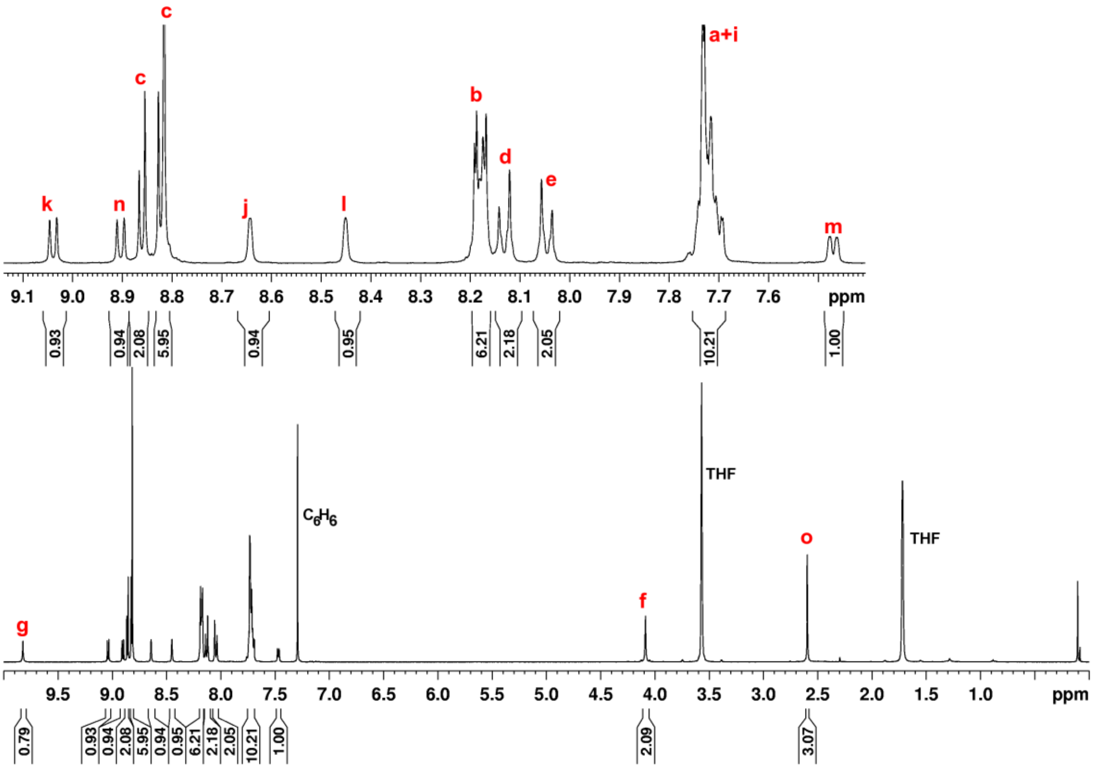

Figure S 2. ^{1}H NMR spectrum of **Dyad 3 Br** (THF-d_8 400 MHz).

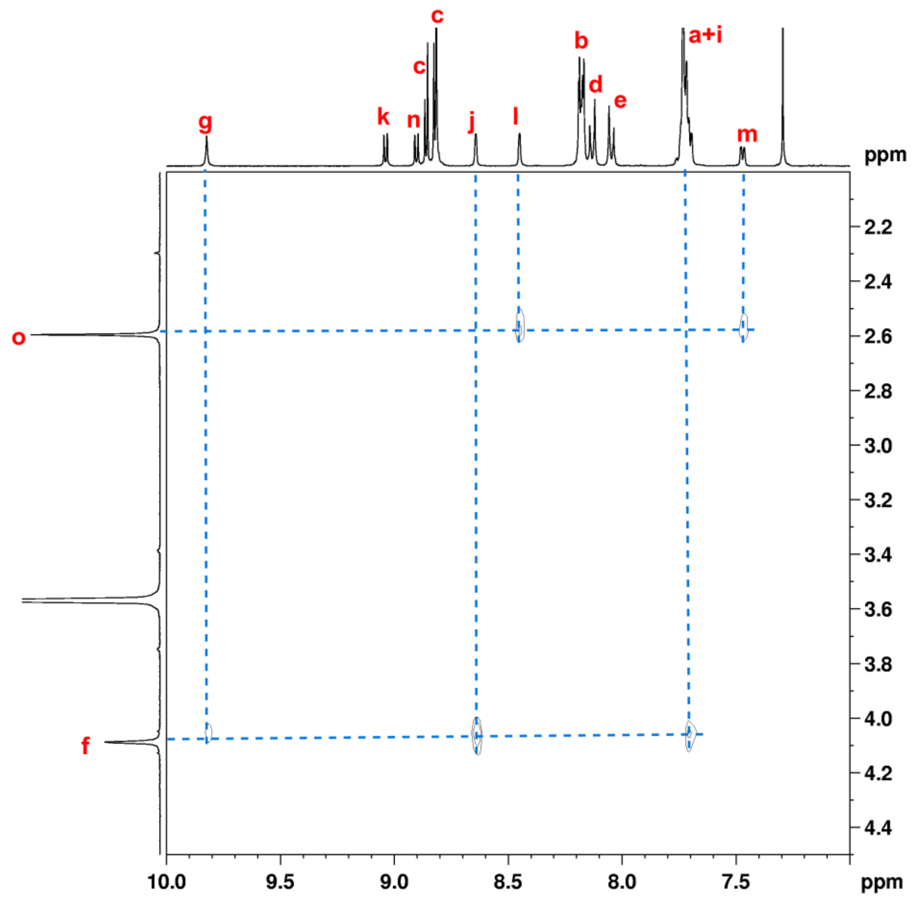

Figure S 3. ^1H-^1H NOESY spectrum of **Dyad 3 Br** (THF-d_8 400 MHz).

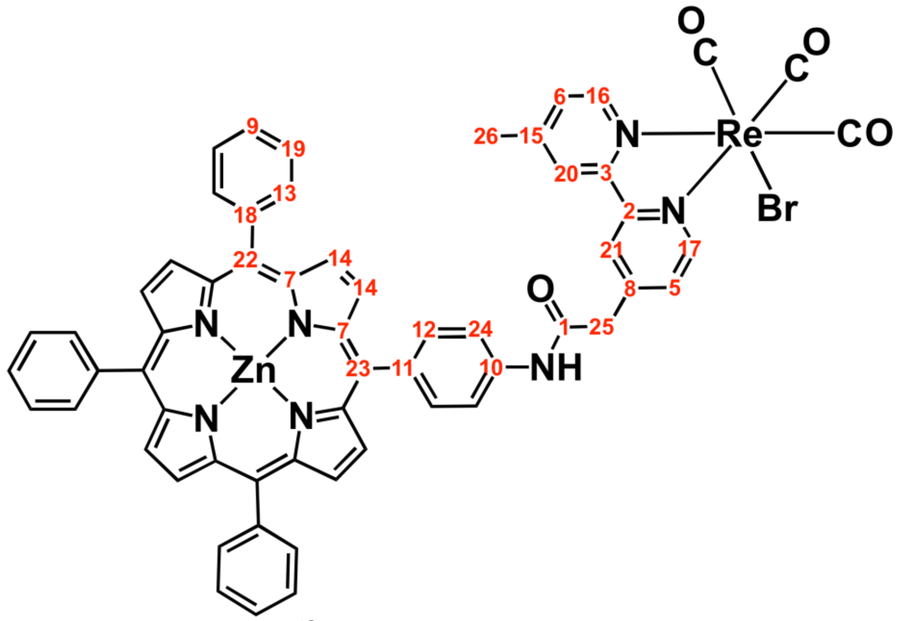

Figure S 4. ^{13}C NMR assignment of **Dyad 3 Br**.

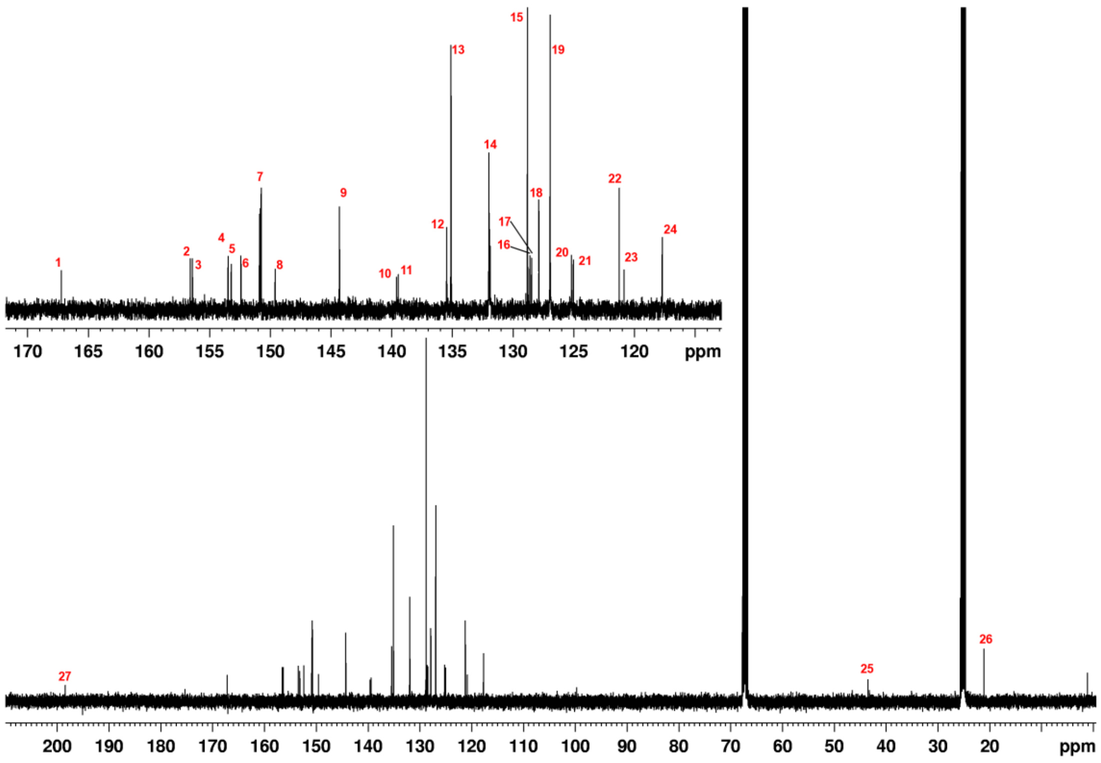

Figure S 5. $^{13}C\{^{1}H\}$ NMR spectrum of **Dyad 3 Br** (THF-d_8 100.6 MHz).

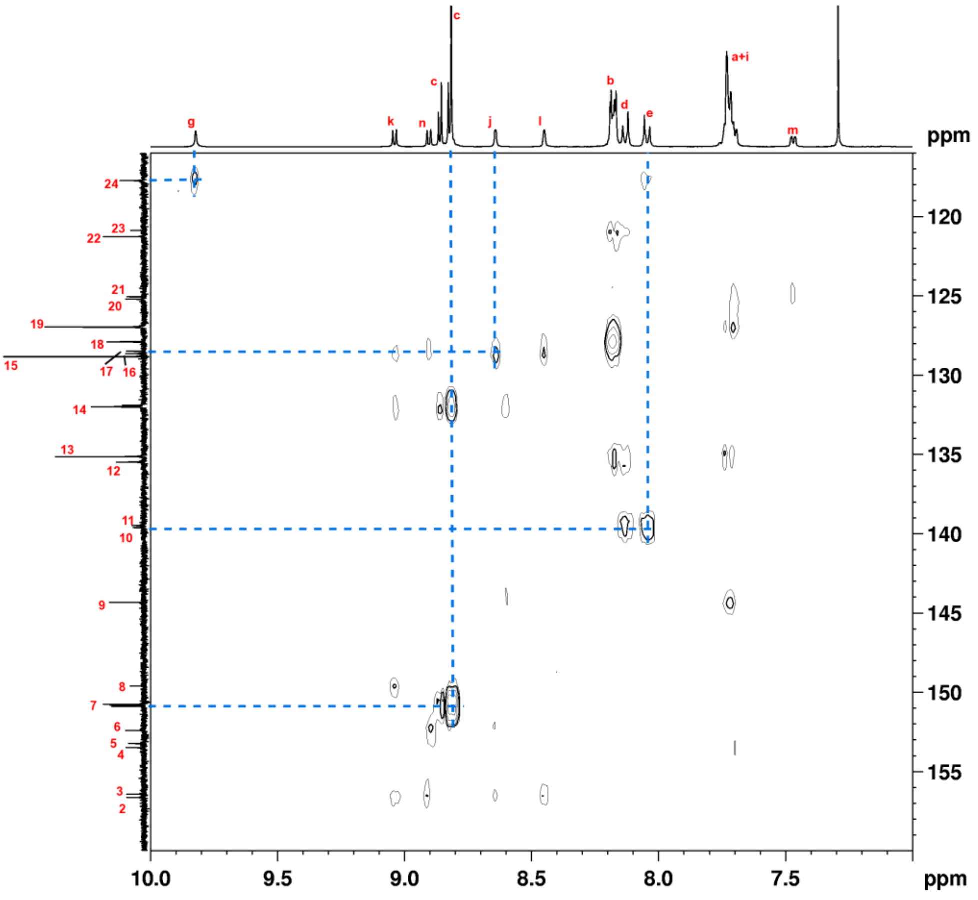

Figure S 6. ^{1}H-^{13}C HMBC NMR spectrum of **Dyad 3 Br** (THF-d_8 400 MHz).

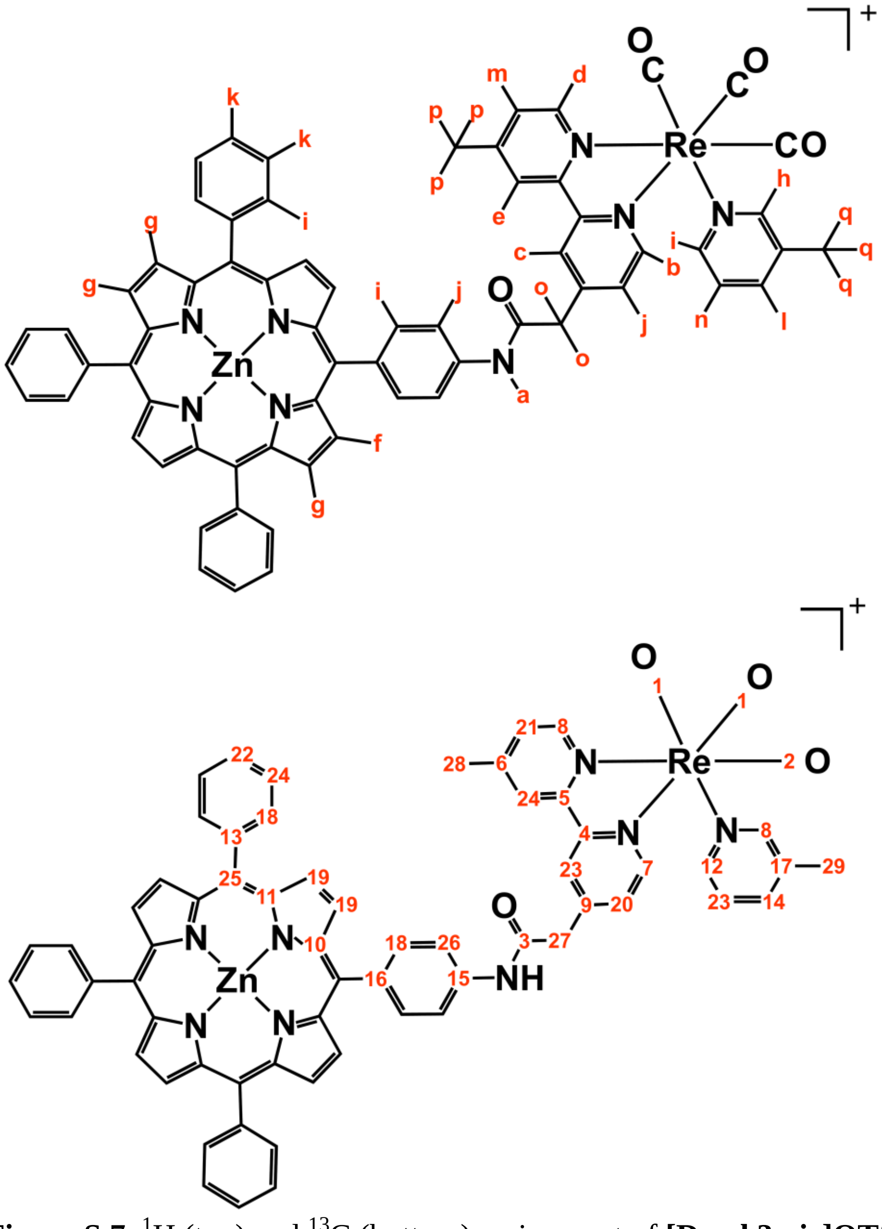

Figure S 7. ^{1}H (top) and ^{13}C (bottom) assignment of **[Dyad 3 pic]OTf**.

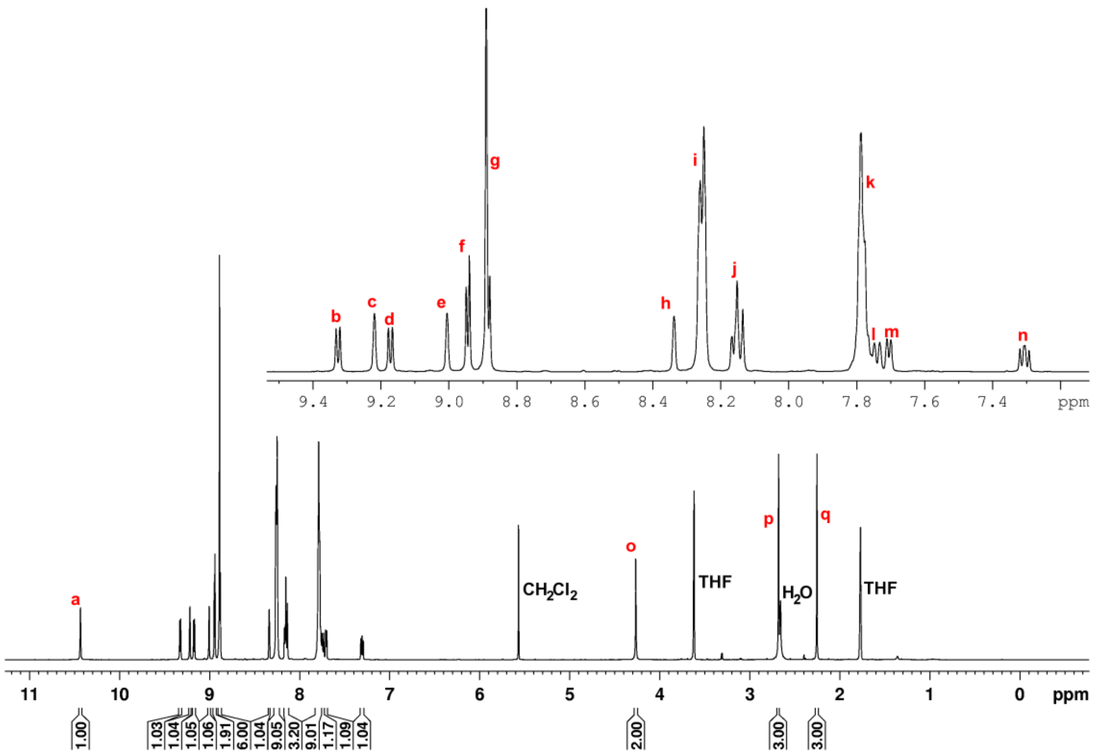

Figure S 8. ^{1}H NMR spectrum of **[Dyad 3 pic]OTf** (THF-d_8 400 MHz).

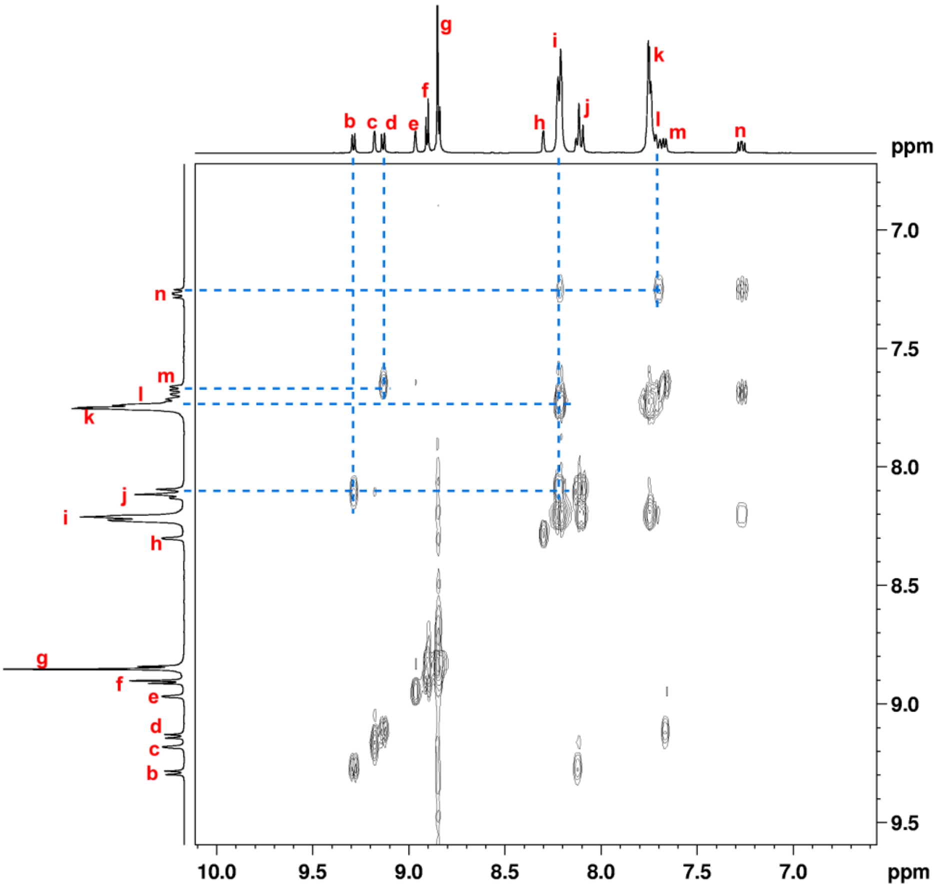

Figure S 9. ^{1}H-^{1}H COSY spectrum of **[Dyad 3 pic]OTf** (THF-d_8 400 MHz).

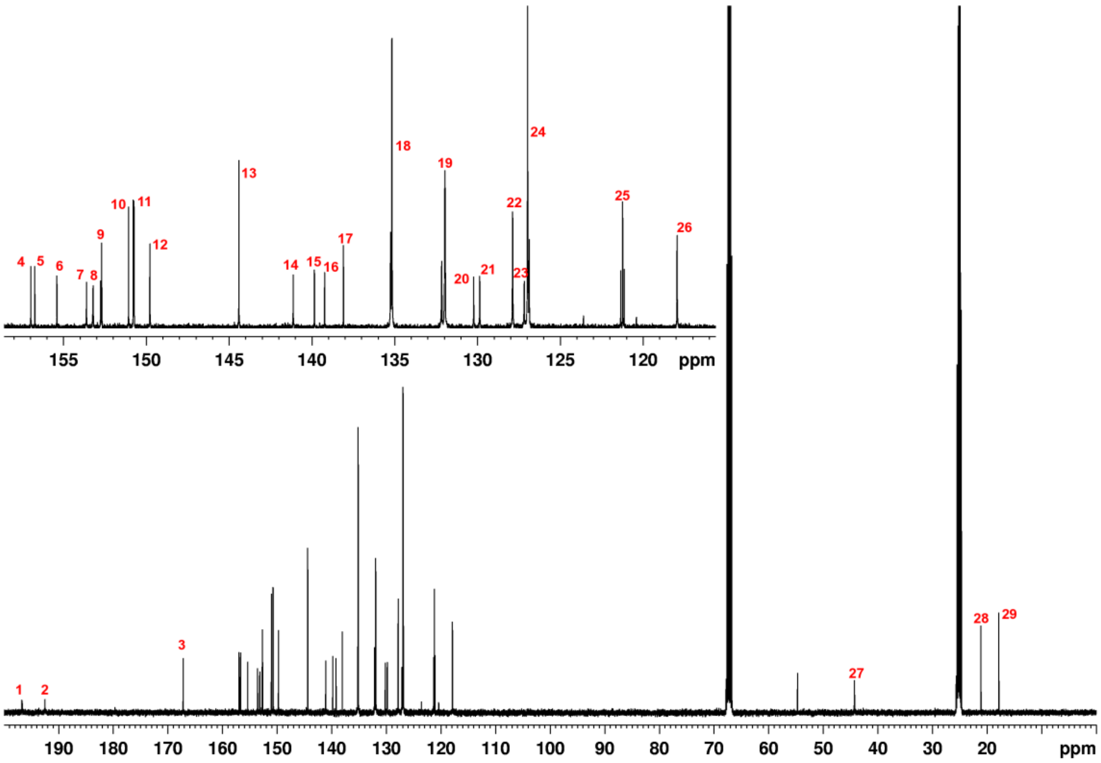

Figure S 10. $^{13}C\{^{1}H\}$ NMR spectrum of **[Dyad 3 pic]OTf** (THF-d_8 100.6 MHz).

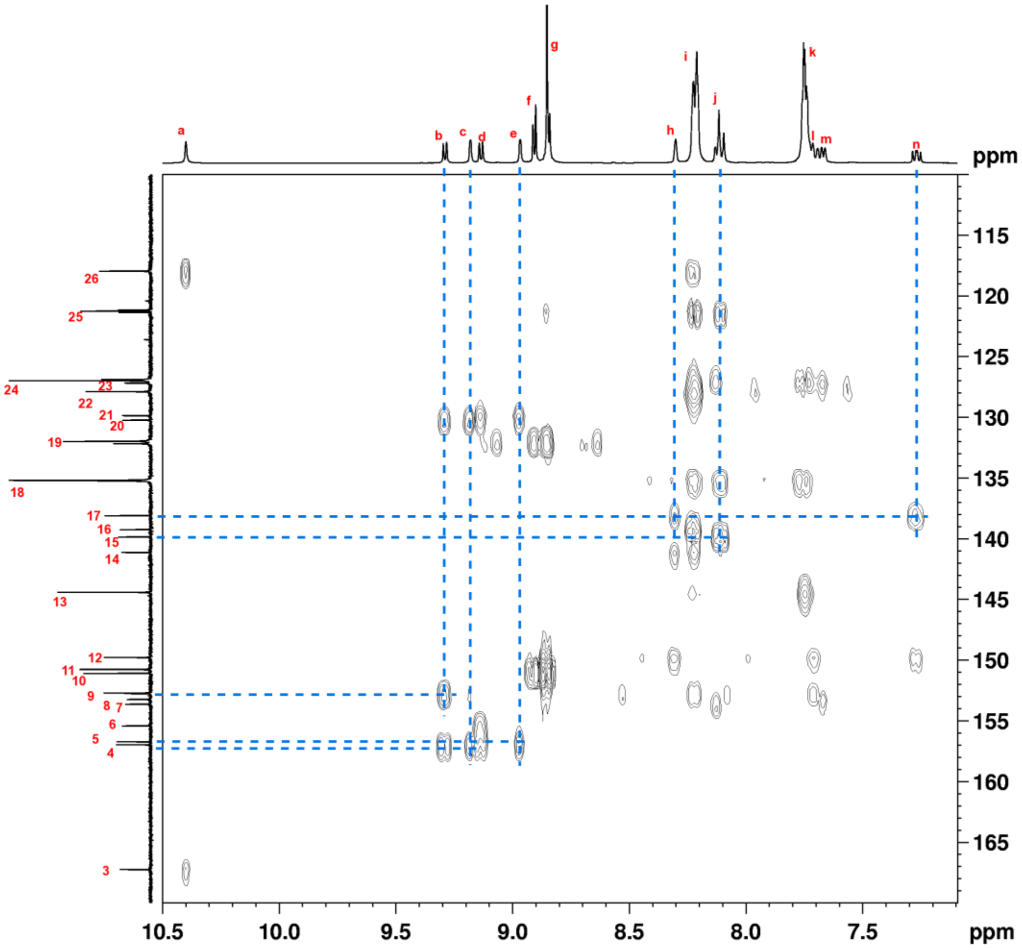

Figure S 11. ^{1}H-^{13}C HMBC NMR spectrum of **[Dyad 3 pic]OTf** (THF-d_8 400 MHz).

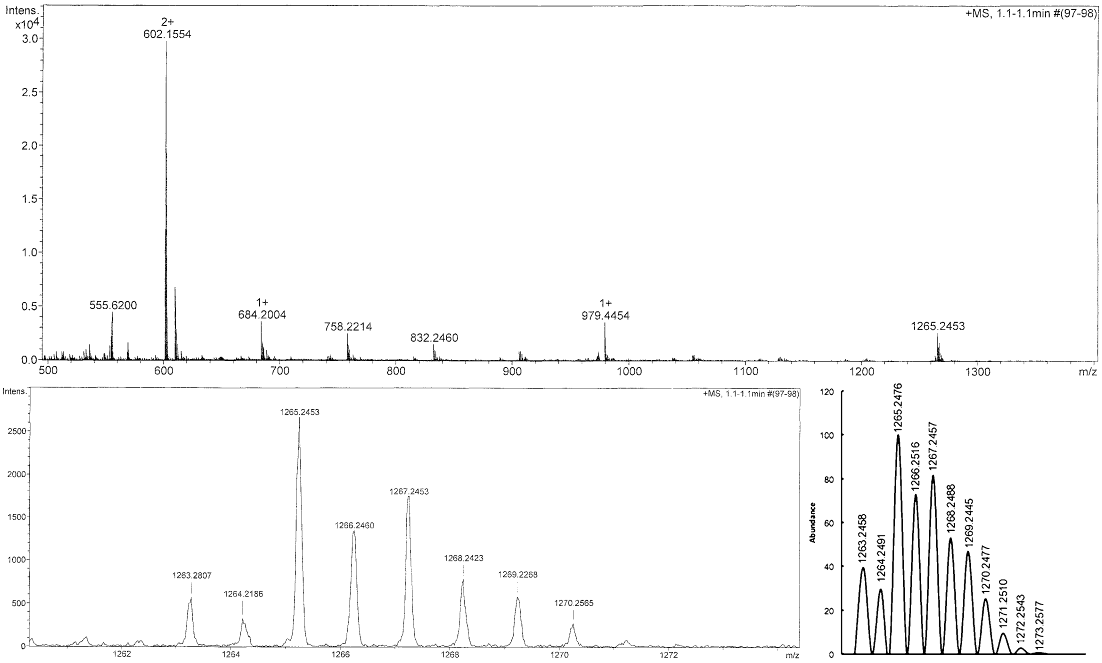

Figure S 12 ESI-MS of **[Dyad 3 pic]OTf**: Top is full mass range, bottom left is expansion of M^+, bottom right is the calculated isotope pattern.

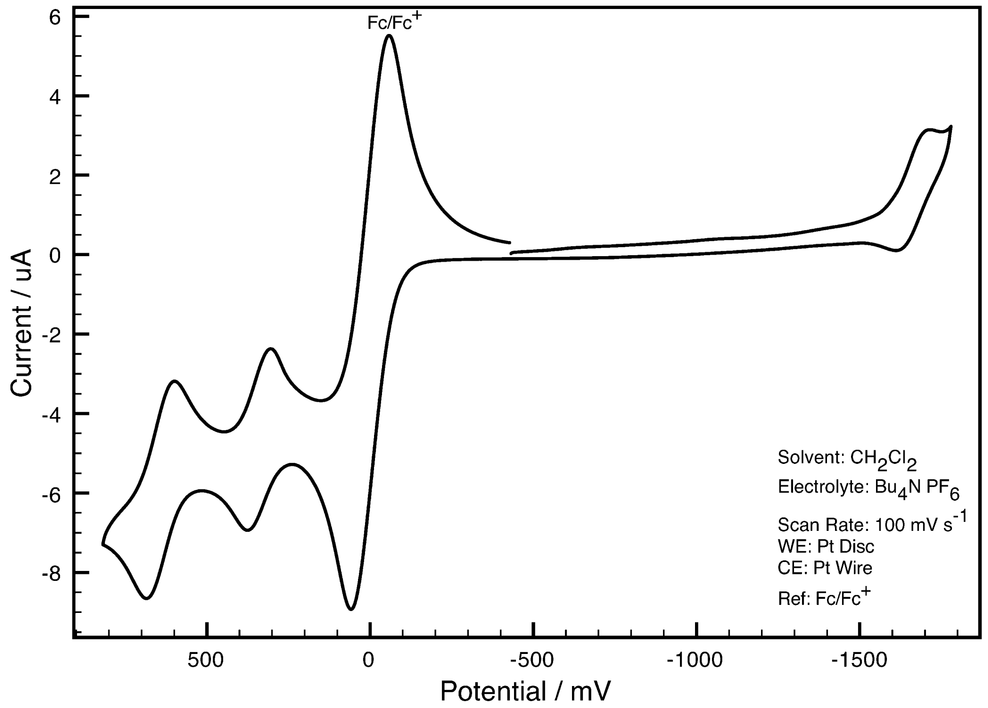

Figure S 13. Cyclic voltammogram of **[Dyad 3 pic]OTf**

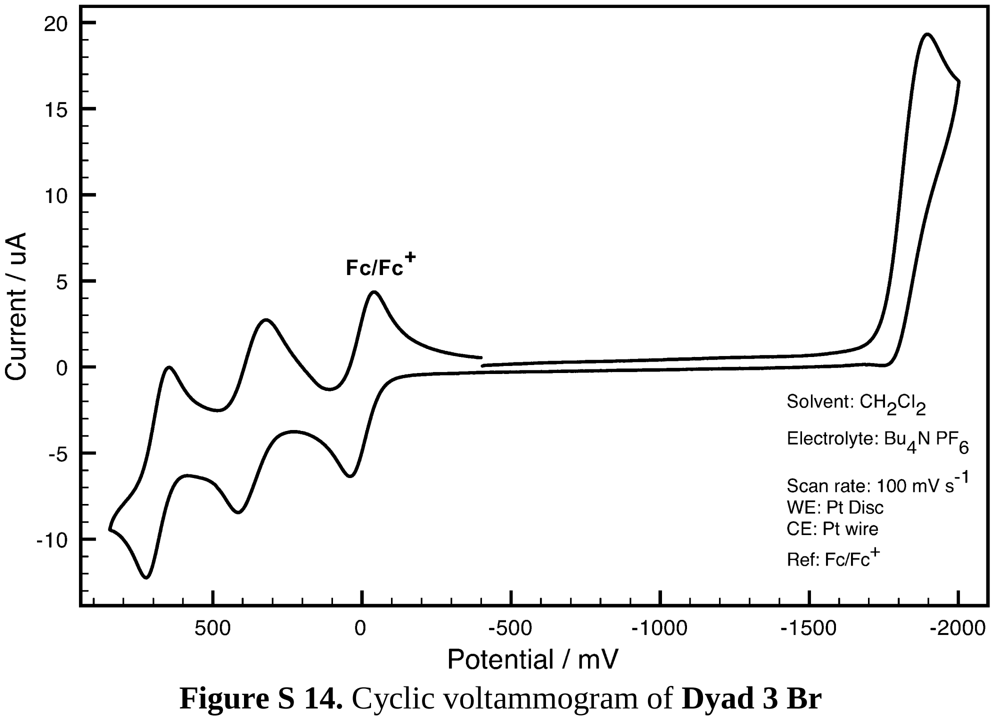

Figure S 14. Cyclic voltammogram of **Dyad 3 Br**

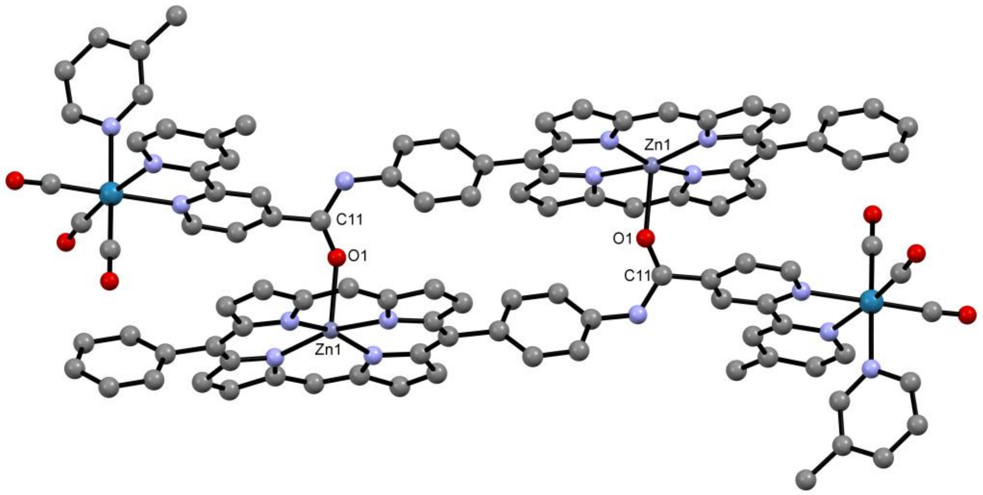

Figure S 15. X-ray crystal structure of **[Dyad 1 pic]PF$_6$**. Hydrogen atoms and 2 porphyrin phenyls, PF_6 counterion and solvent of crystallization omitted for clarity. Selected bond length (Å): Zn(1)-O(1): 2.216(4).

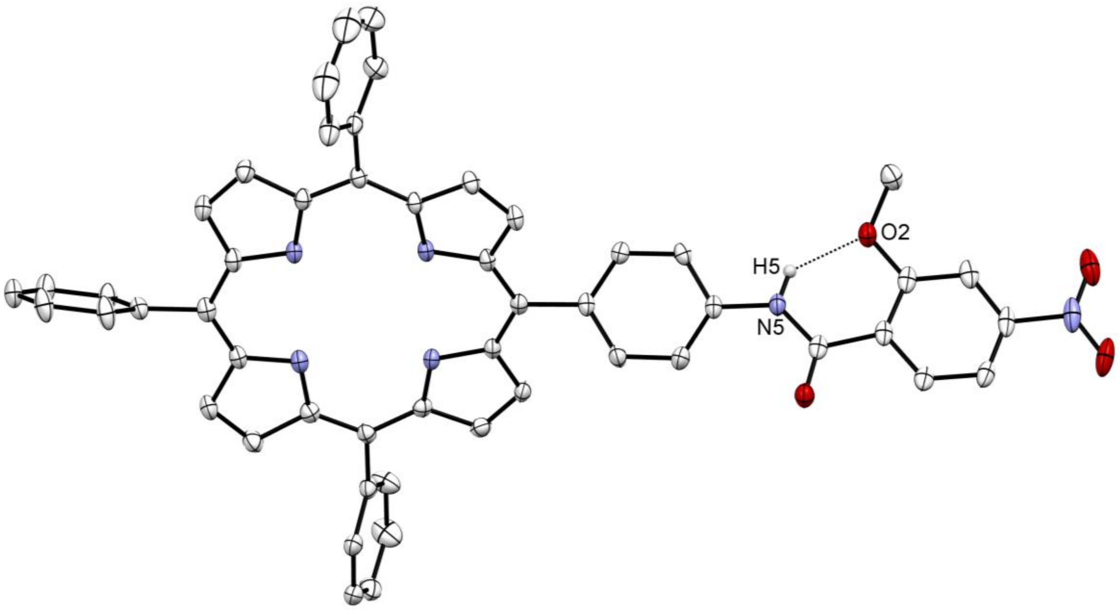

Figure S 16. X-ray crystal structure of 5-[4-[(2-methoxy-4-nitro-phenylcarbonyl)-amino]phenyl]-10,15,20-triphenyl porphyrin. Selected hydrogen atoms and solvent of crystallization omitted for clarity. H(5) was located. Thermal ellipsoids shown with probability of 50%. Selected bond length (Å): O(2)-N(5): 2.658(4).

Table S 1. Crystallographic data for **[Dyad 1 pic][PF$_6$] 2.5 CH$_2$Cl$_2$**

Identification code	rnp1309
Empirical formula	$C_{67.5}H_{49}Cl_5F_6N_8O_4PReZn$
Formula weight	1609.94
Temperature/K	110.00(10)
Crystal system	triclinic
Space group	P-1
a/Å	12.9474(5)
b/Å	15.8804(5)
c/Å	17.3277(6)
α/°	98.752(3)
β/°	92.264(3)
γ/°	106.821(3)
Volume/Å^3	3357.4(2)
Z	2
ρ_{calc}mg/mm^3	1.592
m/mm^{-1}	2.452
F(000)	1602.0
Crystal size/mm^3	0.1612 × 0.091 × 0.0831
2Θ range for data collection	5.7 to 54.2°
Index ranges	$-16 \leq h \leq 16, -20 \leq k \leq 20, -22 \leq l \leq 18$
Reflections collected	25307
Independent reflections	14802[R(int) = 0.0307]
Data/restraints/parameters	14802/25/880
Goodness-of-fit on F^2	1.073
Final R indexes [I>=2σ (I)]	R_1 = 0.0659, wR_2 = 0.1868
Final R indexes [all data]	R_1 = 0.0809, wR_2 = 0.1986
Largest diff. peak/hole / e Å^{-3}	4.12/-1.78
CCDC number	1406001

Table S 2. Crystallographic data for 5-[4-[(2-methoxy-4-nitro-phenylcarbonyl)-amino]phenyl]-10,15,20-triphenyl porphyrin **0.595 CH_2Cl_2**

Empirical formula	$C_{52.59375}H_{37.1875}Cl_{1.1875}N_6O_4$
Formula weight	859.29
Temperature/K	110.00(10)
Crystal system	orthorhombic
Space group	Fddd
a/Å	23.5964(4)
b/Å	37.3470(6)
c/Å	40.0789(6)
α/°	90.00
β/°	90.00
γ/°	90.00
Volume/Å^3	35319.7(9)
Z	32
ρ_{calc}mg/mm^3	1.293
m/mm^{-1}	0.152
F(000)	14303.4
Crystal size/mm^3	0.1818 × 0.1617 × 0.1159
2Θ range for data collection	5.76 to 50.7°
Index ranges	$-25 \leq h \leq 28$, $-44 \leq k \leq 44$, $-45 \leq l \leq 48$
Reflections collected	26924
Independent reflections	8081[R(int) = 0.0340]
Data/restraints/parameters	8081/6/606
Goodness-of-fit on F^2	1.035
Final R indexes [I>=2σ (I)]	R_1 = 0.0578, wR_2 = 0.1587
Final R indexes [all data]	R_1 = 0.0712, wR_2 = 0.1693
Largest diff. peak/hole / e Å^{-3}	0.85/-1.07
CCDC number	1406000

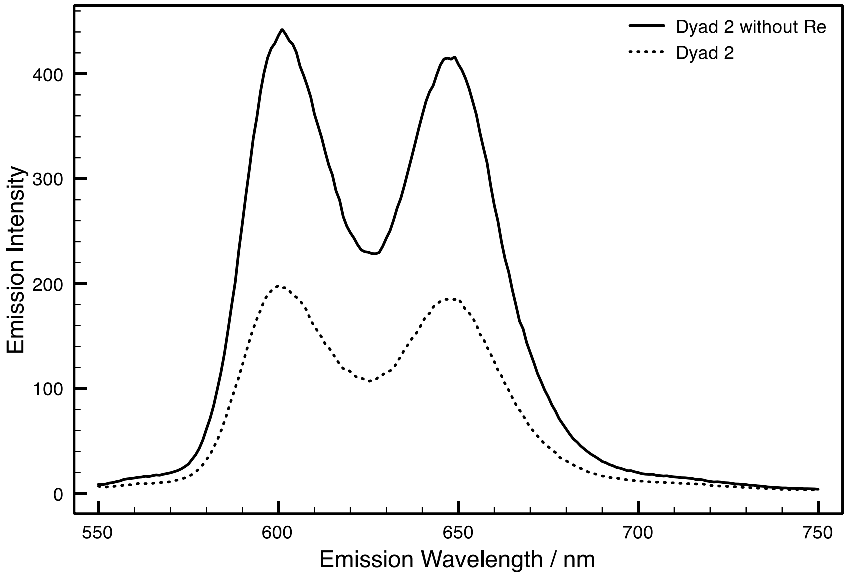

Figure S 17. Emission spectra of **[Dyad 2 pic]OTf** and the Zn porphyrin-bridge-bipyridine ligand of Dyad 2.

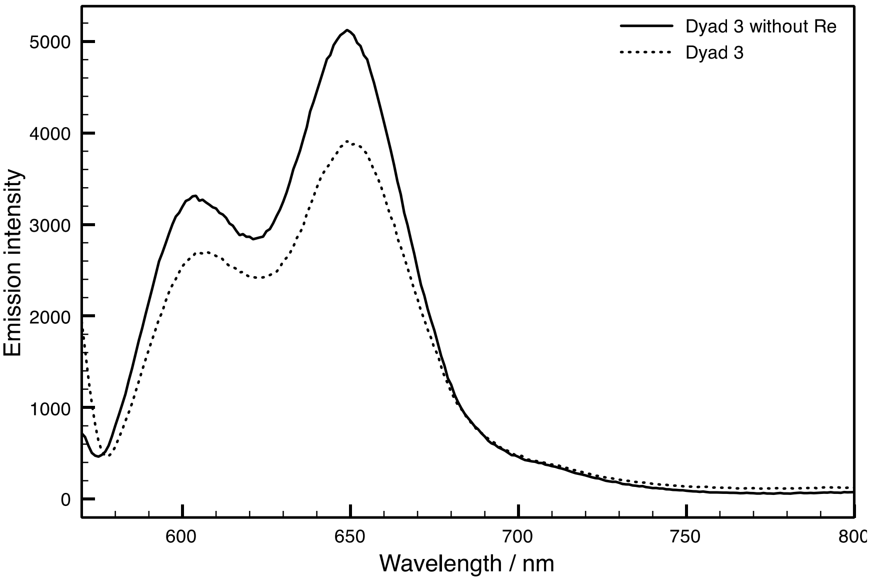

Figure S 18. Emission spectra of **[Dyad 3 pic]OTf** and the Zn porphyrin-bridge-bipyridine ligand of Dyad 3.

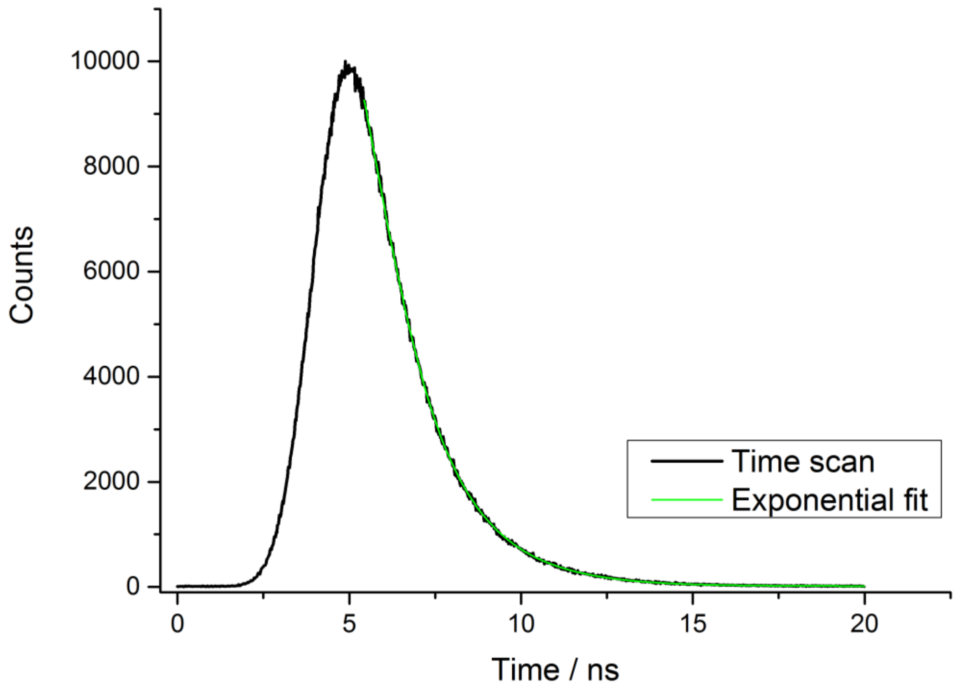

Figure S 19. Emission time scan (black) and fitting (green) for **[Dyad 3 pic]OTf**. λ_{ex} = 560 nm and λ_{em} = 605 nm.

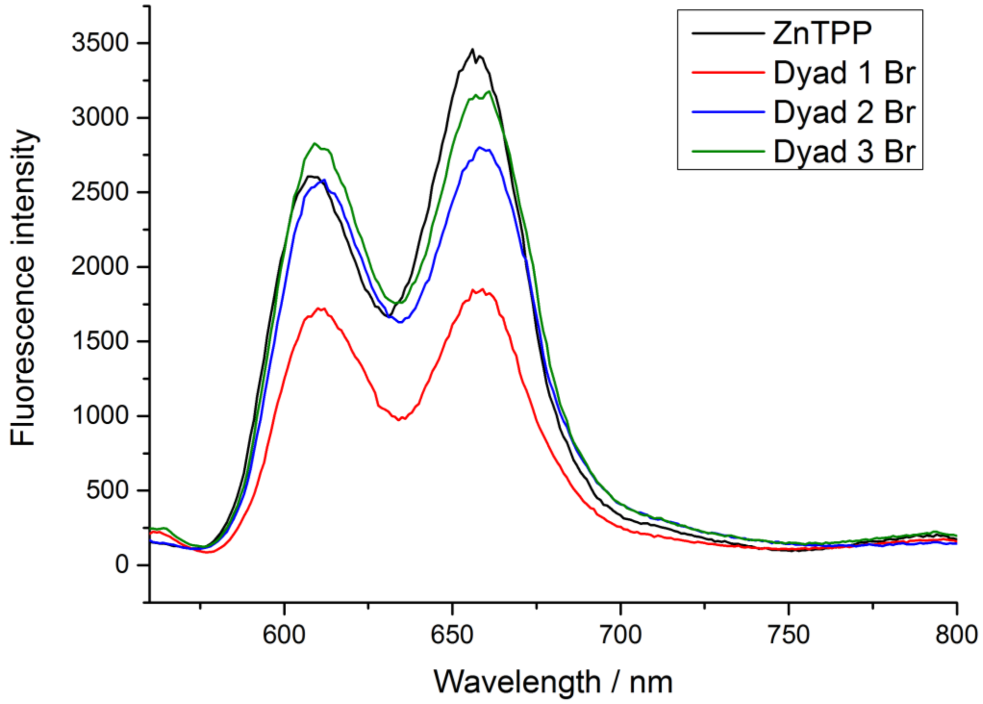

Figure S 20. Emission spectra of **ZnTPP**, **Dyad 1 Br**, **Dyad 2 Br** and **Dyad 3 Br** in THF.

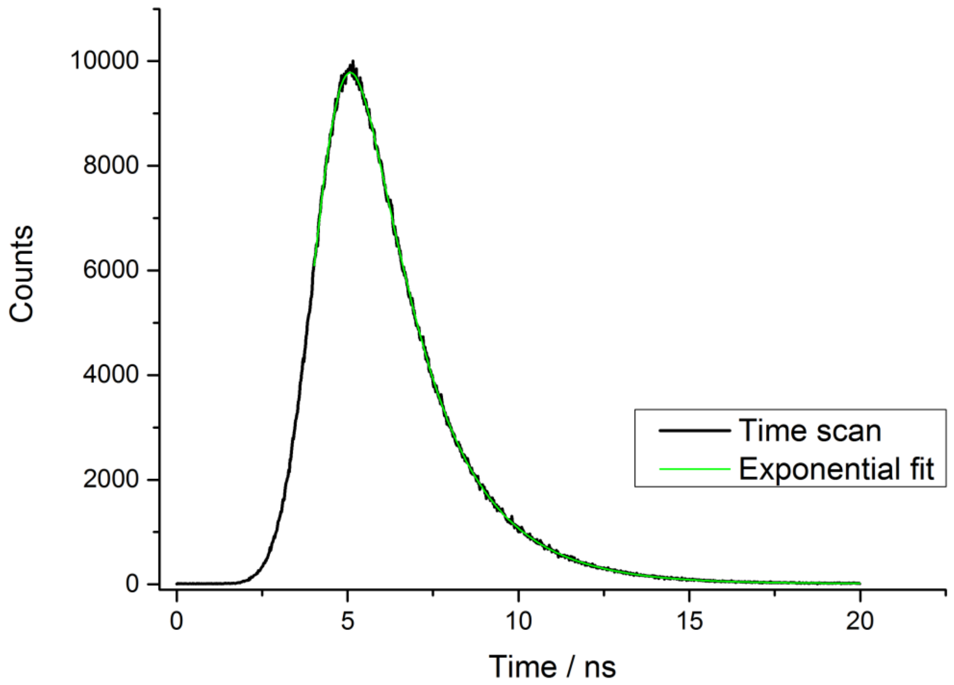

Figure S 21. Emission time scan (black) and fitting (green) for **Dyad 3 Br**. λ_{ex} = 560 nm and λ_{em} = 605 nm.

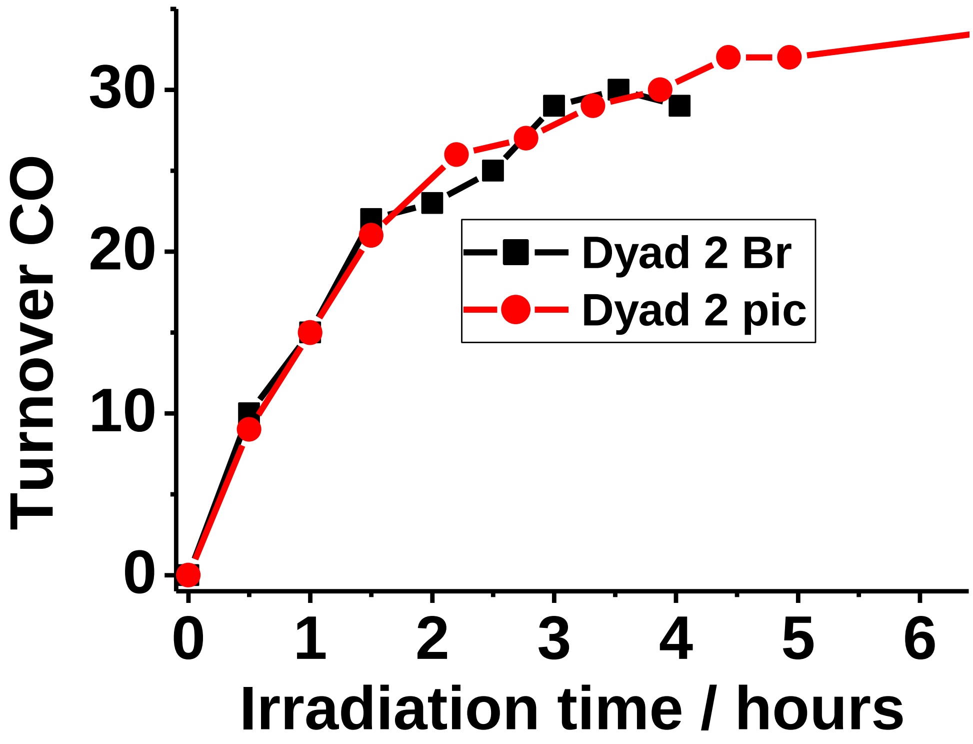

Figure S 22. TON_{CO} for **Dyad 2 Br** and **[Dyad 2 pic]OTf**. In DMF:TEOA 5:1 and with λ > 520 nm irradiation.

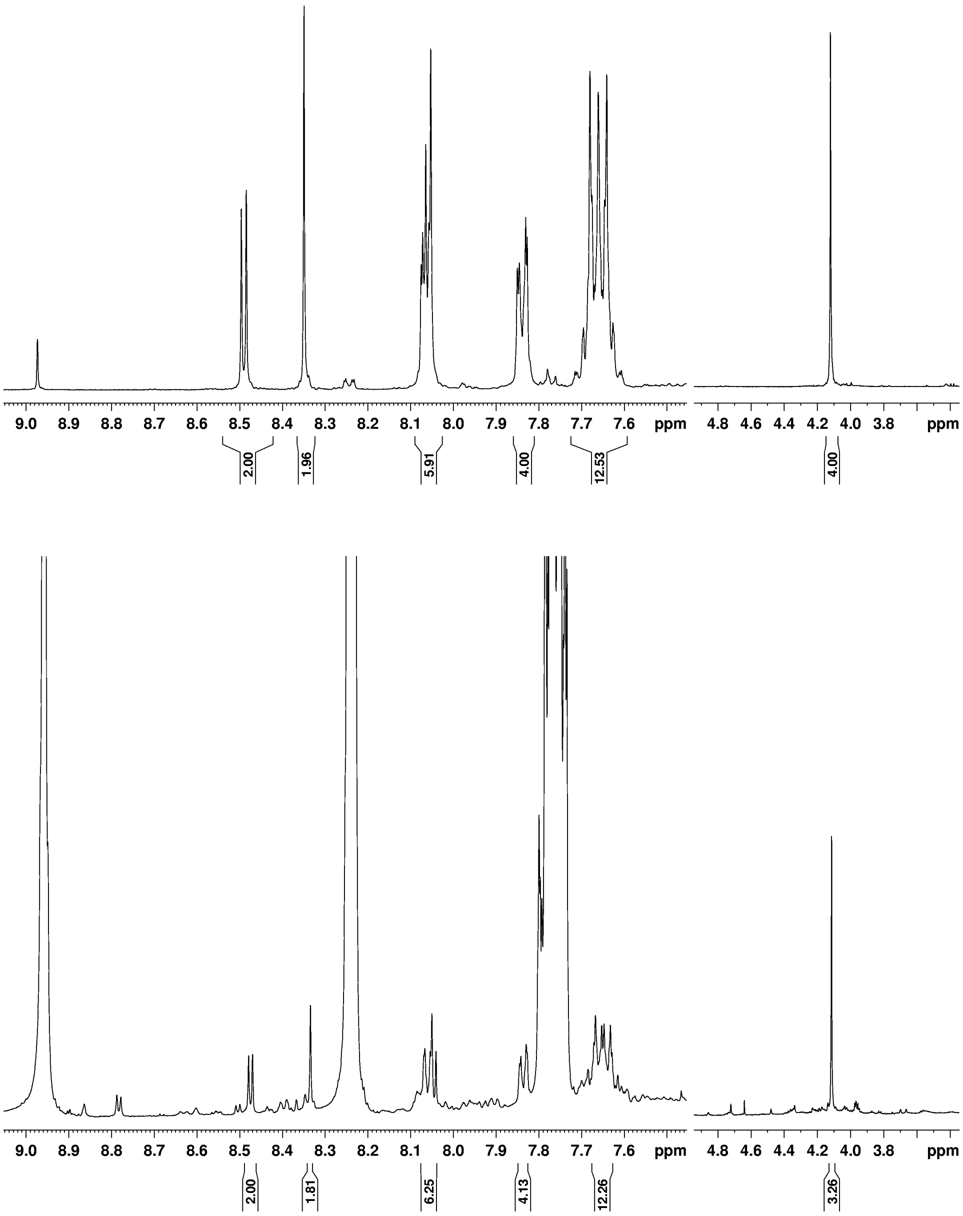

Figure S 23 ^{1}H NMR spectra. Above: authentic zinc tetraphenylchlorin, below: photoreaction mixture of ZnTPP in DMF:TEOA 5:1.

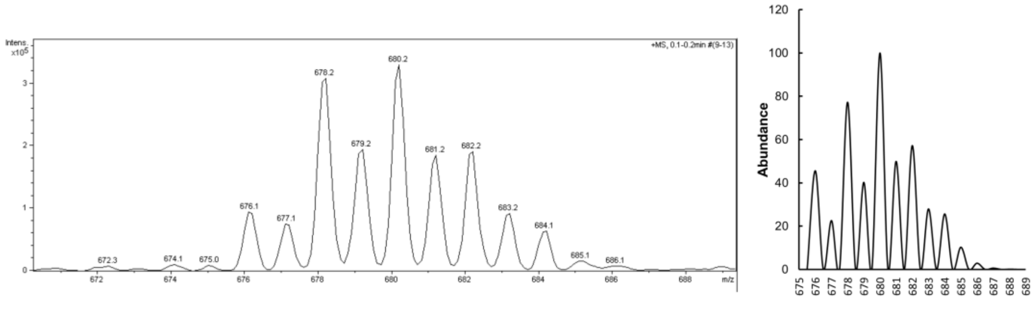

Figure S 24. Left: ESI-MS of ZnTPP in DMF/TEOA after irradiation with λ > 520 nm, right: calculated isotope pattern for ZnTPP, Zn chlorin and Zn isobacteriochlorin superimposed on top of one another in a 1:1:1 ratio.

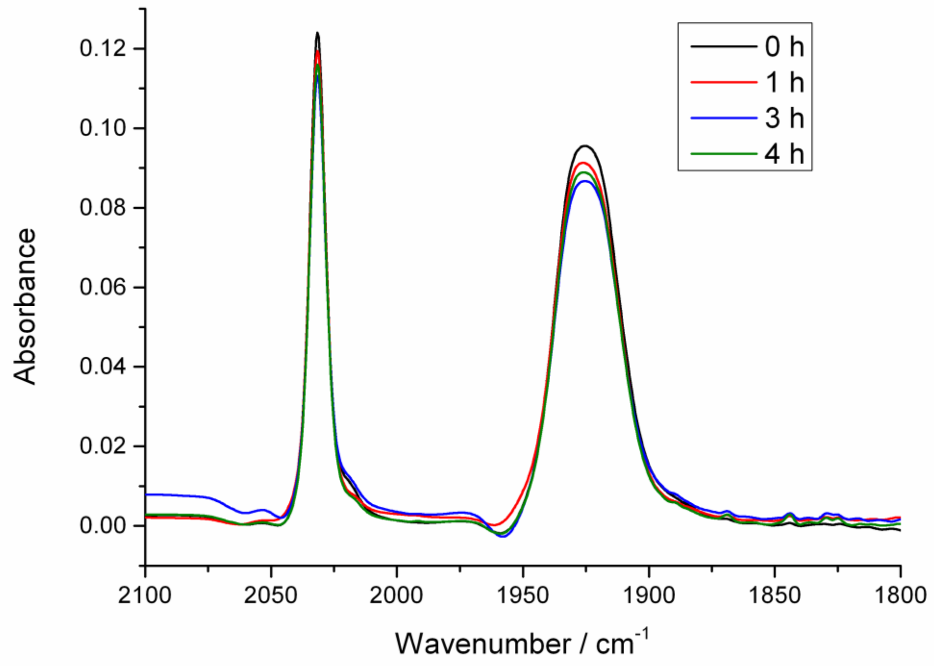

Figure S 25. IR spectra of **[Dyad 2 pic]OTf** following dissolution in DMF at times given.

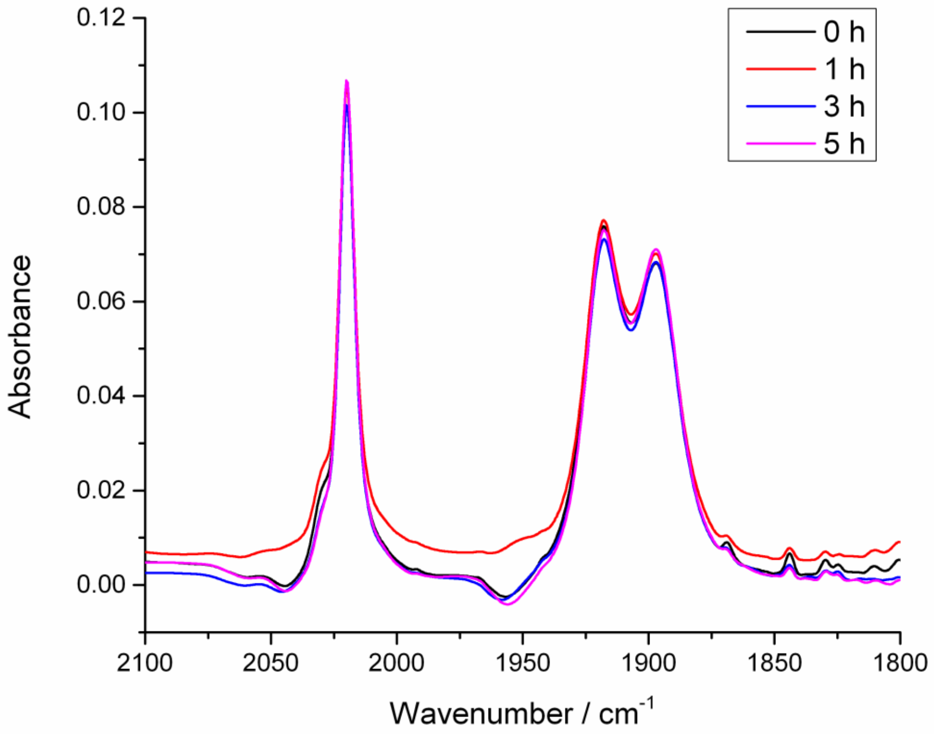

Figure S 26. IR spectra of **Dyad 2 Br** following dissolution in DMF at times given.

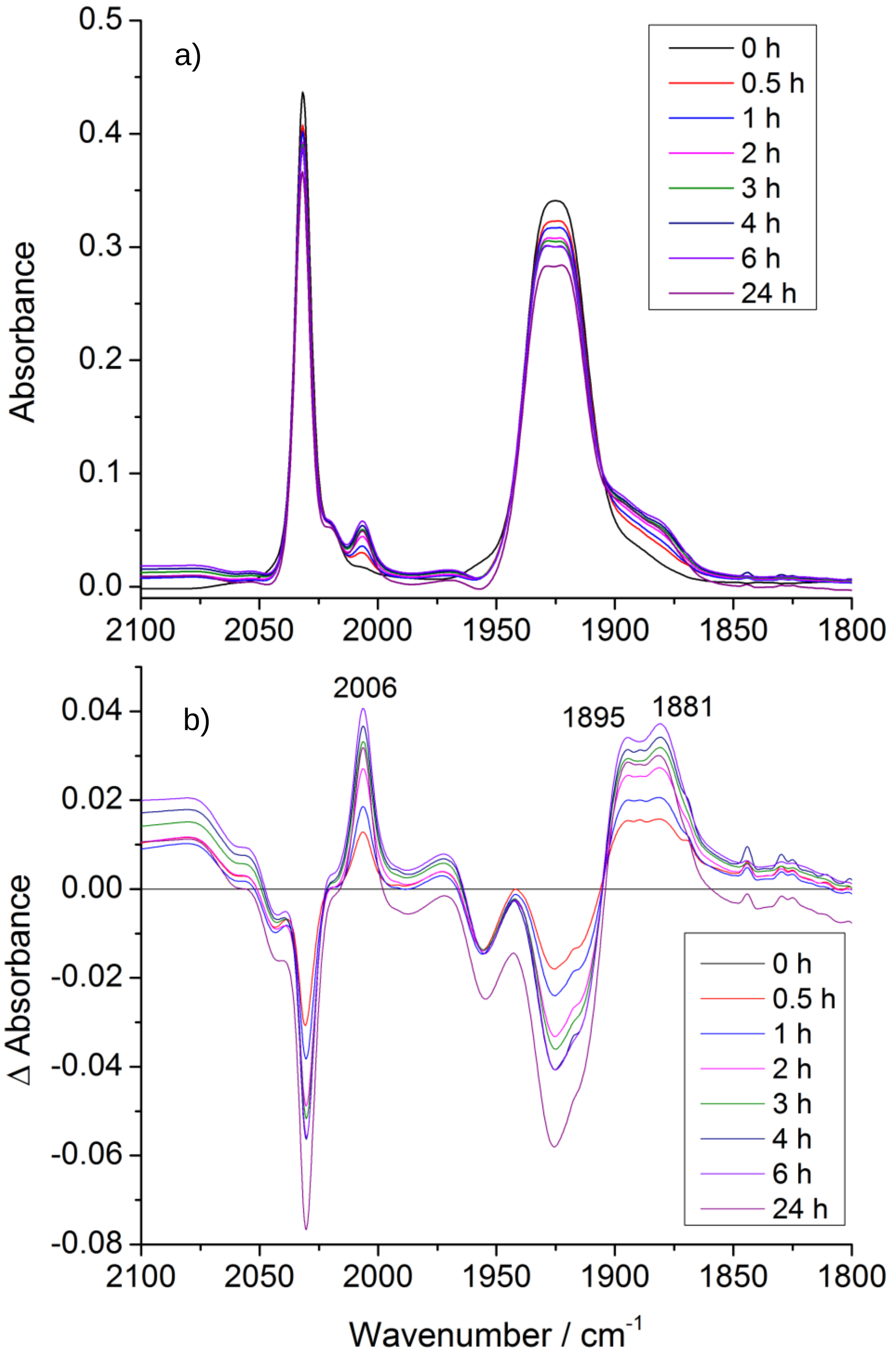

Figure S 27. IR spectra of **[Dyad 2 pic]OTf** in DMF after addition of TEOA (DMF:TEOA 5:1): a) absorption spectra at times after addition of TEOA, b) difference spectra at same times.

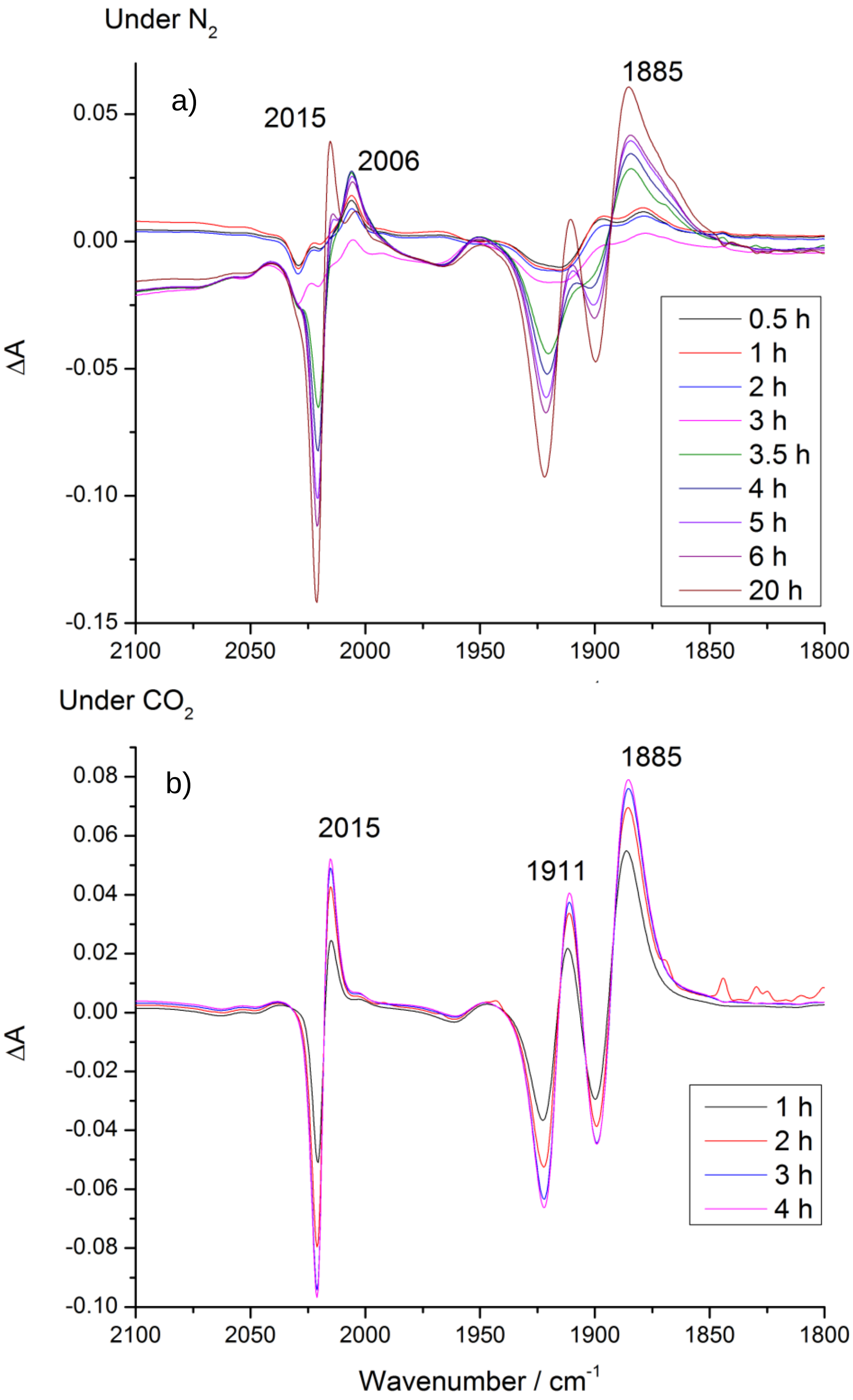

Figure S 28. IR difference spectra of **Dyad 2 Br** in DMF after addition of TEOA (DMF:TEOA 5:1) and with λ > 520 nm irradiation relative to before addition of TEOA: a) under N_2, b) same sample following bubbling of CO_2.

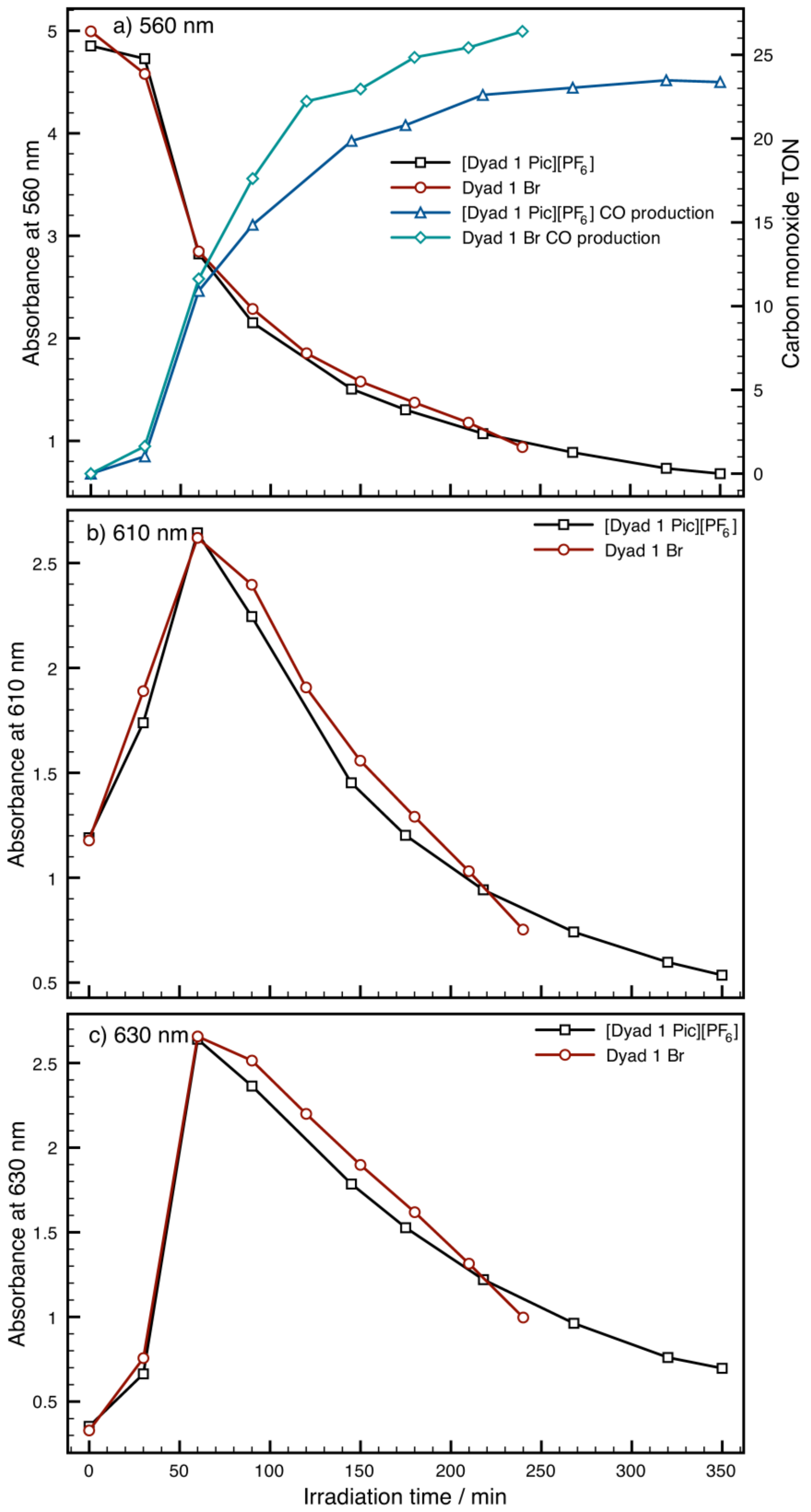

Figure S 29. Absorbance changes in the UV/vis spectra of **Dyad 1 Br** and **[Dyad 1 pic]PF$_6$** during CO_2 reduction: a) changes in signal intensity at 560 nm and turnovers of CO, b) changes in signal intensity at 610 nm, c) changes in signal intensity at 630 nm.
